# Coupled influence of tectonics, climate, and surface processes on landscape evolution in southwestern North America

**DOI:** 10.1038/s41467-022-31903-2

**Published:** 2022-08-01

**Authors:** Alireza Bahadori, William E. Holt, Ran Feng, Jacqueline Austermann, Katharine M. Loughney, Tristan Salles, Louis Moresi, Romain Beucher, Neng Lu, Lucy M. Flesch, Christopher M. Calvelage, E. Troy Rasbury, Daniel M. Davis, Andre R. Potochnik, W. Bruce Ward, Kevin Hatton, Saad S. B. Haq, Tara M. Smiley, Kathleen M. Wooton, Catherine Badgley

**Affiliations:** 1 Lamont-Doherty Earth Observatory, Columbia University in the City of New York, Palisades, NY USA; 2 Department of Geosciences, Stony Brook University, Stony Brook, NY USA; 3 Department of Geosciences, University of Connecticut, Storrs, CT USA; 4 Department of Geology, University of Georgia, Athens, GA USA; 5 School of Geosciences, University of Sydney, Sydney, NSW Australia; 6 Research School of Earth Sciences, The Australian National University, Canberra, Australia; 7 Department of Earth, Atmospheric, and Planetary Sciences, Purdue University, West Lafayette, IN USA; 8 Grand Canyon Conservancy Field Institute, Flagstaff, AZ USA; 9 e4sciences, Sandy Hook, CT USA; 10 Department of Ecology and Evolution, Stony Brook University, Stony Brook, NY USA; 11 Department of Ecology and Evolutionary Biology, University of Michigan, Ann Arbor, MI USA

**Keywords:** Geodynamics, Tectonics, Geophysics, Geomorphology

## Abstract

The Cenozoic landscape evolution in southwestern North America is ascribed to crustal isostasy, dynamic topography, or lithosphere tectonics, but their relative contributions remain controversial. Here we reconstruct landscape history since the late Eocene by investigating the interplay between mantle convection, lithosphere dynamics, climate, and surface processes using fully coupled four-dimensional numerical models. Our quantified depth-dependent strain rate and stress history within the lithosphere, under the influence of gravitational collapse and sub-lithospheric mantle flow, show that high gravitational potential energy of a mountain chain relative to a lower Colorado Plateau can explain extension directions and stress magnitudes in the belt of metamorphic core complexes during topographic collapse. Profound lithospheric weakening through heating and partial melting, following slab rollback, promoted this extensional collapse. Landscape evolution guided northeast drainage onto the Colorado Plateau during the late Eocene-late Oligocene, south-southwest drainage reversal during the late Oligocene-middle Miocene, and southwest drainage following the late Miocene.

## Abstract (web summary)

Cenozoic landscape evolution of southwestern North America remains debated. Here, the authors reconstruct landscape using 4-D numerical models, which can explain extensional collapse and superficial geological record for the Basin and Range Province

## Introduction

The topographic evolution of mountain belts and continental interiors directly reflects the interactions among mantle dynamics, lithosphere tectonics, and climatic and erosional processes. Yet, a long-standing question at the intersection of tectonics, climate, and surface processes (erosion, transport, deposition) is, what roles do tectonics and climate play in driving landscape evolution (along with their impacts on fauna and flora^1–3^)? The 4-D mechanical processes of extensional collapse of topography and transitioning from thickened crustal welts to a flat Moho is not well understood^4–6^. Jackson et al.^7^ argued that thickened crustal roots can remain metastable if dry, but the introduction of water into these lower crustal zones can result in a dramatic weakening. Rosenberg and Handy^8^ showed that significant weakening of continental crust occurs at ~10% melt fraction.

Proposed mechanisms for the Cenozoic landscape evolution in southwestern North America (SWNA) include crustal isostasy^9,10^, dynamic topography^11–13^, or lithosphere tectonics^14–16^, but their relative contributions remain controversial. Recently investigated features of large-scale mantle flow calculations^17–19^ show a pervasive and long wavelength shallow intrusion of eastward asthenospheric flow below the lithosphere of SWNA post-30 Ma that influenced the eastward migration of mafic magmatism there. Taking into account estimates for the extension and shear history over time^20^ and using a mantle thermal model, Bahadori et al.^9^ showed that there was likely a continuous chain of high topography (Nevadaplano and Mogollon Highlands), supported in part by a thickened crustal root, prior to extensional collapse in SWNA. The high gravitational potential energy (GPE) of this mountain chain could have potentially generated deviatoric tensional stresses that were a fundamental driving mechanism for extensional collapse. Bahadori and Holt^10^ found that changing boundary conditions along with the influences of the excess GPE distributions were not sufficient themselves to explain the apparent timing and location of topographic collapse in SWNA. In addition to these factors, effective viscosities of the lithosphere are required to be dramatically lowered during times of topographic collapse^10^. Bahadori and Holt^10^ argued for temperature, hydration, and the presence of melt as primary factors for the weakening of the lithosphere during the slab rollback history in SWNA. However, their results involved the response of vertical averages for lithosphere rheology and therefore did not address the 3-D crustal and upper mantle response.

**Fig. 1:**
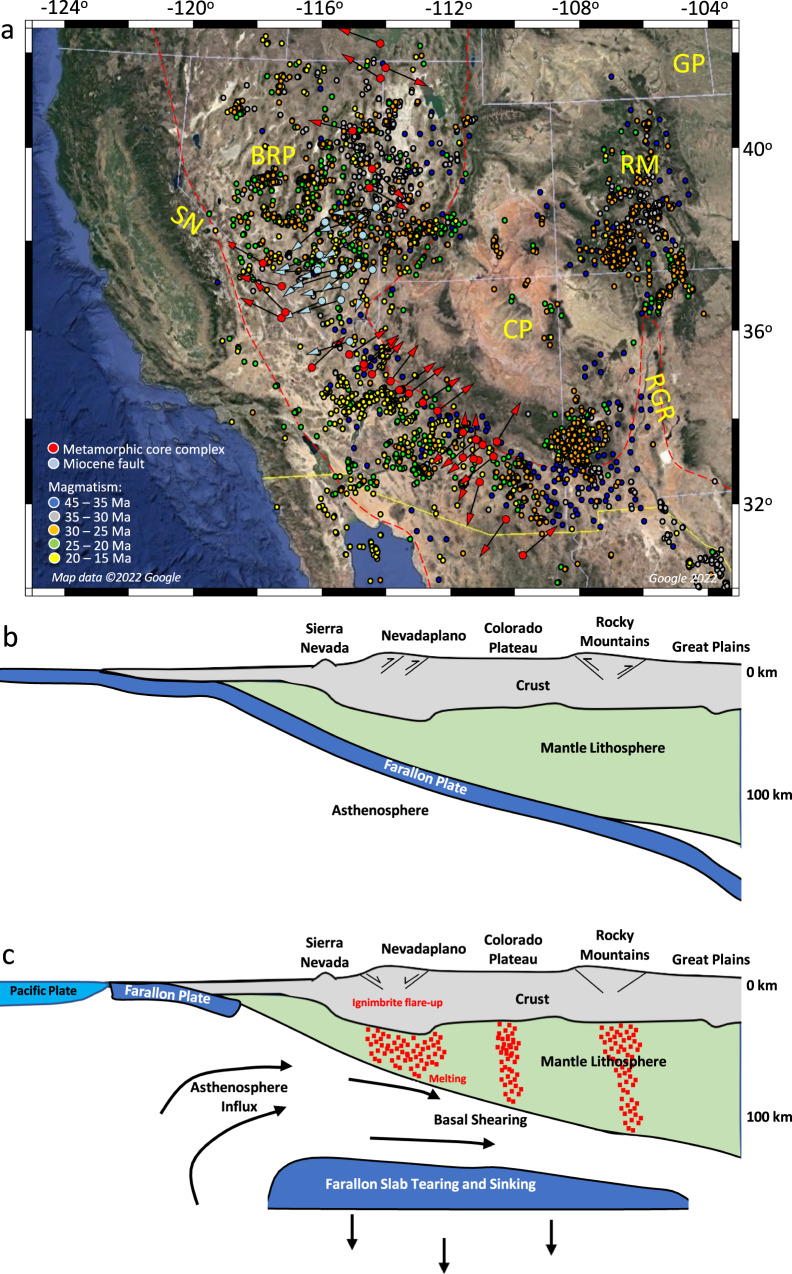
Tectonic history and relation of mantle processes to surface geologic activity of southwestern North America. **a** Satellite image of southwestern North America showing the location of the Basin and Range Province (BRP), area surrounded by red dashed line, Colorado Plateau (CP), Rio Grande Rift (RGR), Great Plains (GP), and Sierra Nevada (SN) from Google Earth (2022). Red spots are the present-day locality of metamorphic core complexes, and red arrows represent vergence of Cordilleran metamorphic core complexes from Bahadori and Holt^10^. Light blue spots are the present-day locality of Miocene faults, and light blue arrows represent Miocene fault trends from Bahadori and Holt^10^. Color-coded dots represent distribution of magmatism at different times following the slab rollback from Bahadori and Holt^10^. **b** A representation of the Laramide orogeny showing the southwestern margin of North America with subducted Farallon Slab in contact with North American Plate at around 60 Ma modified from Humphreys^26^. **c** A representation of the Farallon Slab tearing, partial melting, ignimbrite flare-up, magmatism, and crustal extension in southwestern North America at 35 Ma modified from Humphreys^26^. The map images were created by authors using: www.soest.hawaii.edu/gmt/ and www.paraview.org/.

Following the Sevier-Laramide phases of contraction, crustal shortening, and mountain building, associated with the history of normal and flat subductions and terrane accretion^21,22^, SWNA experienced a dramatic phase of lithospheric extension, resulting in the present-day Basin and Range Province (BRP) (Fig. 1a). The flat subduction of the Farallon Slab has been suggested to have truncated the overriding continental lithosphere^23^ and introduced water into the North American (NA) lithospheric mantle^15,24,25^. Lithosphere foundering of the Farallon Slab that started at around 50 Ma^21,26^ triggered volcanism and initiated an ignimbrite flare-up by influx of hot asthenosphere underneath the overriding NA Plate during the Eocene, Oligocene, and Miocene^22,27,28^ (Fig. 1b, c).

A period of extension was, for the most part, initiated following the collision of the East Pacific Rise with the Farallon subduction margin beginning at 30 Ma^29,30^. Crustal stretching and topographic collapse within areas of highest extension resulted in significant exhumation of deep crustal rocks in metamorphic core complexes (MCCs)^31,32^ of SWNA (Fig. 1a). In the northern BRP, the slab rollback progressed from northeast to southwest during the Eocene and Oligocene (~55–20 Ma) and resulted in MCC ages that young towards the south^33^. In the south (southern Arizona and New Mexico, Northern Mexico) the slab rollback progressed from east to west during the late Eocene to early Miocene (~35–17 Ma), resulting in MCCs that young towards the west^33^. The youngest MCCs, developed during the Miocene (~17–5 Ma), coincide with the western edges of the southwestern Great Basin within southern Nevada and eastern California (Fig. 1a).

Although significant progress has been made toward quantifying the dynamics of the plate boundary zone in SWNA, a complete description of the time-dependent driving forces behind this complex strain history has remained contentious. Considerable controversy surrounds how and when the topography and crustal structure evolved in SWNA, along with the initiation and migration of fluvial drainage systems^34–37^. A full understanding of the lithospheric deformation mechanisms in space and time, and how they relate to the topographic collapse and surface uplift history, requires a comprehensive dynamic approach, which includes not only interactions between mantle convection and lithosphere but also the contribution from effective crustal body forces, climate, and surface processes.

In this work, we show that recent advances in the field have made it possible to couple all these processes together, and we argue here that numerical simulation is a powerful method that can help resolve the complex history of the lithosphere dynamics (deformation) and landscape evolution in SWNA. Therefore, this study uses numerical models to simulate all the processes mentioned above simultaneously to better understand the source of extension within the BRP, topographic collapse, MCC formation, and evolution of a deeply incised Grand Canyon.

We reconstruct the landscape history by considering major driving factors that could have affected lithosphere evolution in SWNA since the late Eocene. These driving factors include: (1) the effects of gravitational body forces caused by gravity acting on the 3-D structure of the lithosphere, (2) the effects of traction field and temperature evolution associated with mantle convection below the NA lithosphere caused by the coupling of mantle flow with lithosphere, and (3) the effects of surface erosion and mass redistribution caused by the coupling of lithosphere with climate and surface processes. Thus, our fully coupled 4-D thermo-mechanical model of lithosphere evolution accounts for the depth-dependent body force effects generated by surface and Moho topography, while also accounting for a layered nonlinear visco-plastic rheology for crust and upper mantle, as well as incorporating effects of mantle convection at the base and mass wasting at the top. Here, we show that our geodynamic approach provides the opportunity to pull all the key elements together to quantify the driving mechanisms for the deformation history and that also places important features addressed separately in previously published works into a coherent time-dependent context (e.g., magmatism, rheology, paleo-elevation and paleo-crustal structure, metamorphic core complexes, structural data for faults and dykes, paleo-climate, sedimentary packages, paleo-drainage paths).

## Results

### Formulating a fully coupled 4-D model

The synthesis of decades of field studies and high-definition geophysical data, makes SWNA an ideal place to study the influences of tectonics and climate on landscape evolution. Refined tomography models from EarthScope USArray^38–40^, refined crustal thickness models^41^, palinspastic restorations^9^, plate reconstructions^42^, and time-dependent tomography-based mantle flow calculations^17–19^ now provide the opportunity to tackle the temporal evolution of mantle and lithosphere interactions, and to use these to predict topography, climate, and drainage.

**Fig. 2:**
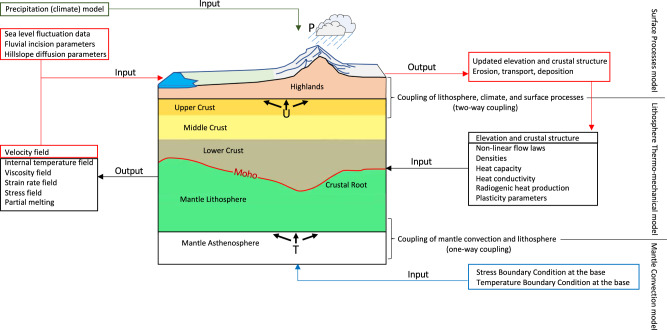
Schematics of the coupling between mantle convection, lithosphere dynamics, climate, and surface processes. A representation of the model setup, boundary conditions, inputs, and outputs of simulations. ‘T’ is the sub-lithosphere tractions from mantle convection. ‘U’ is the 3-D displacement field for the upper crust. ‘P’ is the precipitation model.

We evaluate quantitatively the lithosphere deformation and landscape evolution (Fig. 2) by simultaneously incorporating the dynamic influences within the lithosphere from estimates of paleo-topography, paleo-crustal thickness, time-dependent thermal boundary condition based on the palinspastically restored history of magmatism following slab rollback, reconstructed paleo-climate models, as well as reconstructed mantle convection and its associated sub-lithosphere tractions.

### Stress boundary condition inferred from tomography-based mantle flow model

**Fig. 3:**
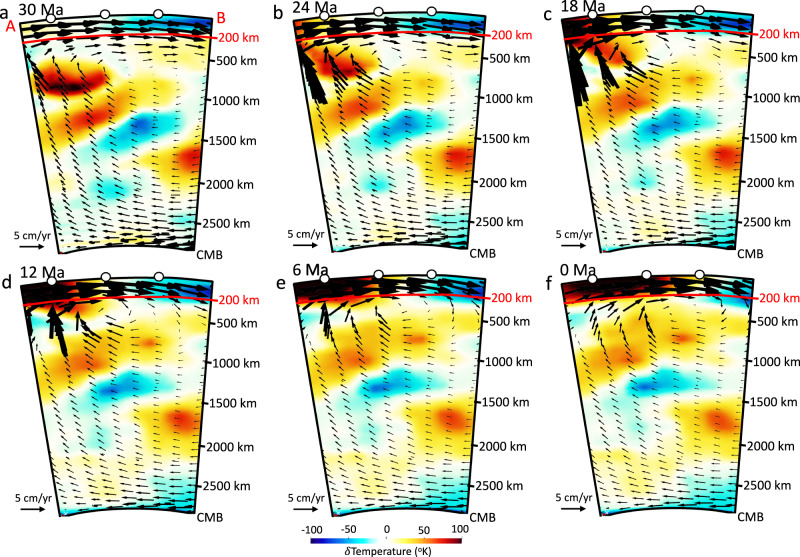
Evolution of mantle thermal structures beneath the Basin and Range Province since the late Eocene. **a**–**f** Radial cross-sections of the reconstructed mantle temperature variations from surface to core–mantle boundary (CMB) along the line A–B at latitude 37^o^N in Fig. 4. Superimposed on these cross-sections are the corresponding mantle flow velocity vectors. The vectors are extracted from global flow field in the mantle’s no-net rotation (NNR) frame of reference along a cross section fixed relative to North America. Panels show landward intrusion of hot anomalies within the Basin and Range Province. The map images were created by authors using: www.soest.hawaii.edu/gmt/ and www.paraview.org/.

**Fig. 4:**
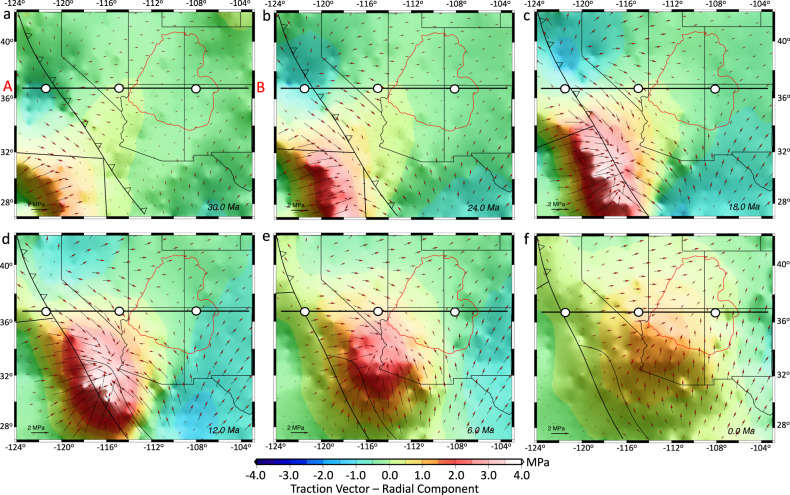
Evolution of the traction field associated with mantle convection at 200 km depth. **a**–**f** Vectors represent horizontal components of the traction vector field at 200 km depth, and contours represent the radial component of the traction vector field at 30, 24, 18, 12, 6, and 0 Ma, respectively. The closed red line indicates the present-day edge of the Colorado Plateau. The map images were created by authors using: www.soest.hawaii.edu/gmt/ and www.paraview.org/.

We use the finite-element software ASPECT^43–45^ to simulate the temporal evolution of global mantle flow from backward mantle convection to the late Eocene (“Methods”). To perform the simulations, we use the global density distribution model TX2008^17,40^ that represents present-day mantle and lithospheric density variations, inferred from joint seismic-geodynamic inversions by considering mineral physical constraints on the scaling of seismic shear-wave speed to density^17,40^. The convection simulation incorporates Newtonian rheology with a viscosity profile that is constrained by global joint inversions of convection-related surface observables and data associated with the response of the Earth to ice-age surface mass loading^46^. This viscosity profile is labeled ‘V2’ in Moucha et al.^17^. We define a free-slip boundary at the surface and core–mantle boundary of the model. The resulting model of global mantle convection shows the motion of a warm mantle upwelling towards the interior of the SWNA since the late Eocene (Fig. 3, Supplementary Movie 1) and provides estimates of spatial and temporal variations in deviatoric stress tensors and the associated traction field at different depths. We use the sub-lithosphere tractions at 200 km depth (Fig. 4, Supplementary Movie 2) as boundary conditions for the 4-D thermo-mechanical model of the lithosphere. The lithosphere model will then maintain the defined stress field at the base by adjusting the flow across the border (boundary) of our finite element mesh. This is how the one-way coupling of mantle convection and lithosphere is implemented.

### Lithosphere dynamics and tectonic model

**Fig. 5:**
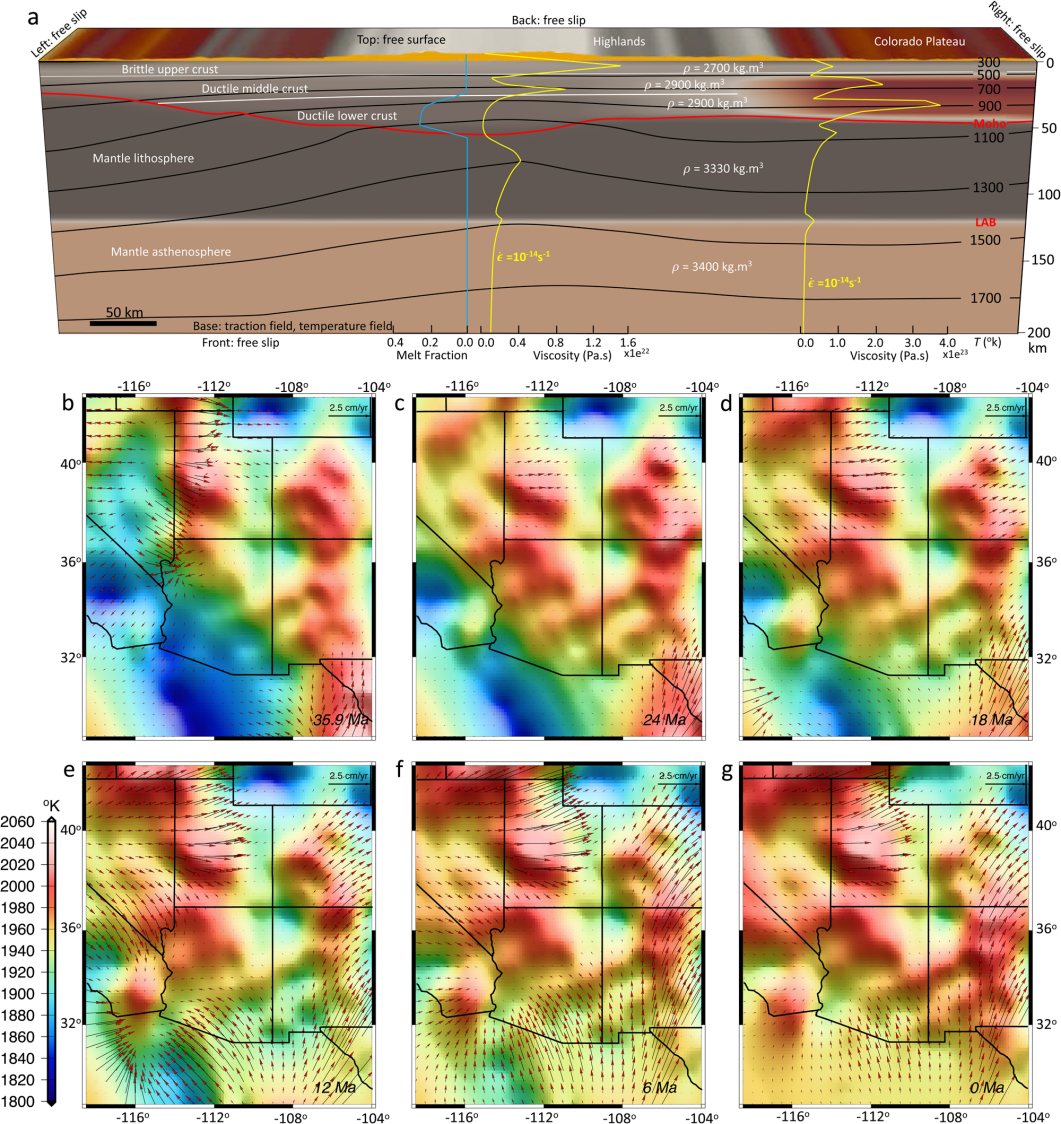
Model setup and boundary conditions for the 4-D thermo-mechanical model of the lithosphere. **a** Model geometry, parameters and boundary conditions, temperature, and strength as a function of depth. The yellow graphs represent the defined starting viscosities based on a constant strain rate of 1e^−14^ s^−1^. LAB: lithosphere-asthenosphere boundary; T: temperature; ρ: density. **b**–**g** Temperature variations at 200 km depth used in thermo-mechanical modeling of the lithosphere at 35.9, 24, 18, 12, 6, and 0 Ma, respectively. Red arrows represent kinematic field associated with sub-lithosphere tractions from mantle convection at 200 km depth. Black lines are the present-day state boundaries. The map images were created by authors using: www.soest.hawaii.edu/gmt/ and www.paraview.org/.

We use the model of Bahadori and Holt^10^ to generate a starting 3-D crustal and upper mantle structure for the lithosphere in SWNA at the late Eocene. The strength of the crust and mantle are represented by a non-Newtonian visco-plastic rheology (Methods). In addition to solving the equilibrium equations for viscous–plastic flow within the lithosphere in three dimensions, we also solve for the thermal evolution of the model. The mechanical and thermal systems are coupled through the temperature dependence of viscosity and density and are solved sequentially during each model time step (0.1 Myr). The initial temperature field for the model is defined based on a linear initial geotherm with a thermal boundary condition at the top of the model that is the absolute temperatures of 273 K and a time-dependent laterally varying temperature field at the base of the model (Fig. 5a). The spatial and temporal evolution of temperature at the base of the lithosphere (Fig. 5b–g, Supplementary Movie 3), inferred from the palinspastically restored positions of volcanism within the time-dependent extensional framework^9,10^, together with spatial and temporal evolution of stresses at the base of the lithosphere (Fig. 4), inferred from the mantle convection model, are important constraints in our simulations. The thermo-mechanical model of the lithosphere produces estimates of velocities within the upper crust that are used as a forcing condition in the surface processes model. This is how the two-way coupling of lithosphere and surface processes is implemented. In our fully coupled lithosphere and surface processes models, while interactions between erosion, tectonics, and climate form, modify, or destroy geomorphic features, the transfer of mass that results from erosion and sedimentation can, to some extent, affect isostasy and the mechanical behavior of the lithosphere by producing localized strain. Moreover, the simulated surface uplift in our models include the exhumation rates associated with erosion that happens at the surface^47^.

### Surface processes model

In our surface processes simulation, with external forcings such as motion of the upper crust, sea-level fluctuations, and reconstructed paleo-climate (precipitation) models (Supplementary Figs. 1 and 2) (“Methods”), we quantify the relative importance of tectonics, climate, and surface processes for landscape evolution in SWNA. Thus, spatial and temporal changes in erosion rate, sediment distribution and accumulation, as well as updated paleo-elevation estimates are the main outputs of our surface processes simulations, calculated in each 0.1 Myr time increment (Fig. 2). The direct comparison between model outputs and the palinspastically restored history of sediment accumulation from Macrostrat^48^ (post-late Eocene) provides an important test of the viability of the 4-D thermo-mechanical model for both its timing and mechanisms for the landscape evolution of SWNA.

### Body force and mantle flow influences on lithospheric deviatoric stresses

**Fig. 6:**
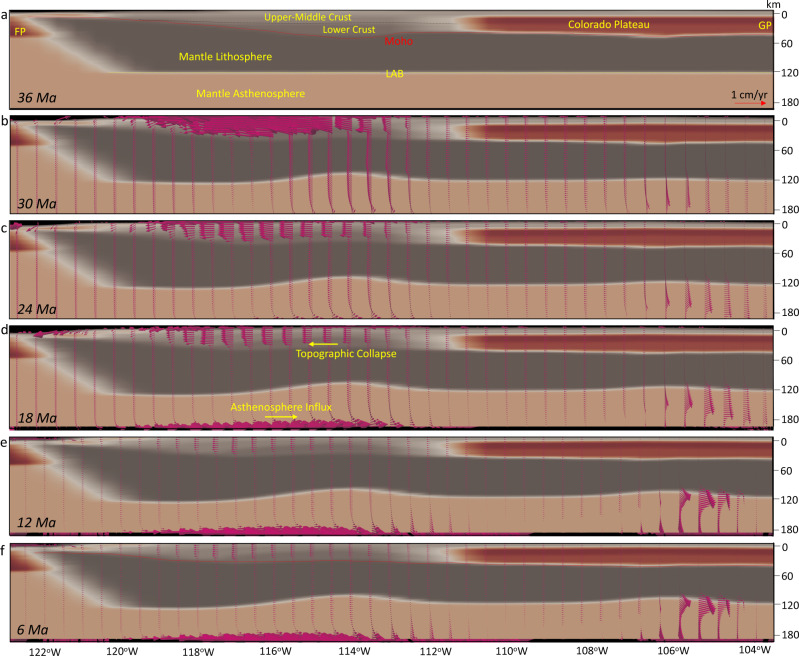
Lithosphere dynamics and tectonic model achieved from 4-D thermo-mechanical model of the North American lithosphere. **a**–**f** Vertical cross-sections of the 3-D lithosphere and mantle asthenosphere at 37°N showing the evolution of a layered 3-D crust for the Basin and Range Province, Colorado Plateau, and Great Plains (GP) in southwestern North America at 36, 30, 24, 18, 12, and 6 Ma, respectively. Red vectors represent the magnitude of velocities at different depths through time. The solid red line represents the evolution of the Moho boundary, and the yellow dashed line represents the lithosphere-asthenosphere boundary (LAB) at the onset of simulation. FP: Farallon Plate.

**Fig. 7:**
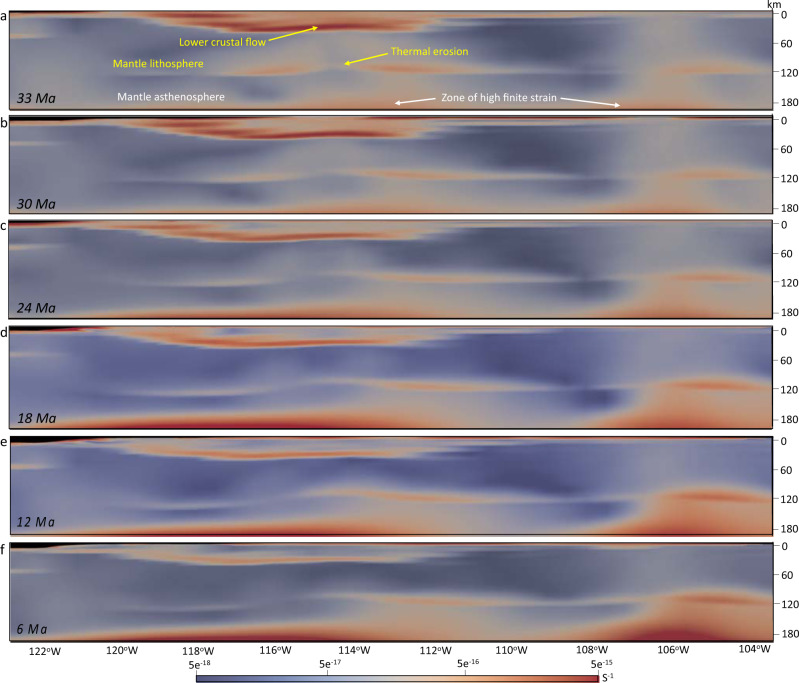
Lithosphere dynamics and tectonic model achieved from 4-D thermo-mechanical model of the North American lithosphere. **a**–**f** Vertical cross-sections of the 3-D lithosphere and mantle asthenosphere at 37°N showing the evolution of finite strain rate within the Basin and Range Province, Colorado Plateau, and Great Plains in southwestern North America at 33, 30, 24, 18, 12, and 6 Ma, respectively.

**Fig. 8:**
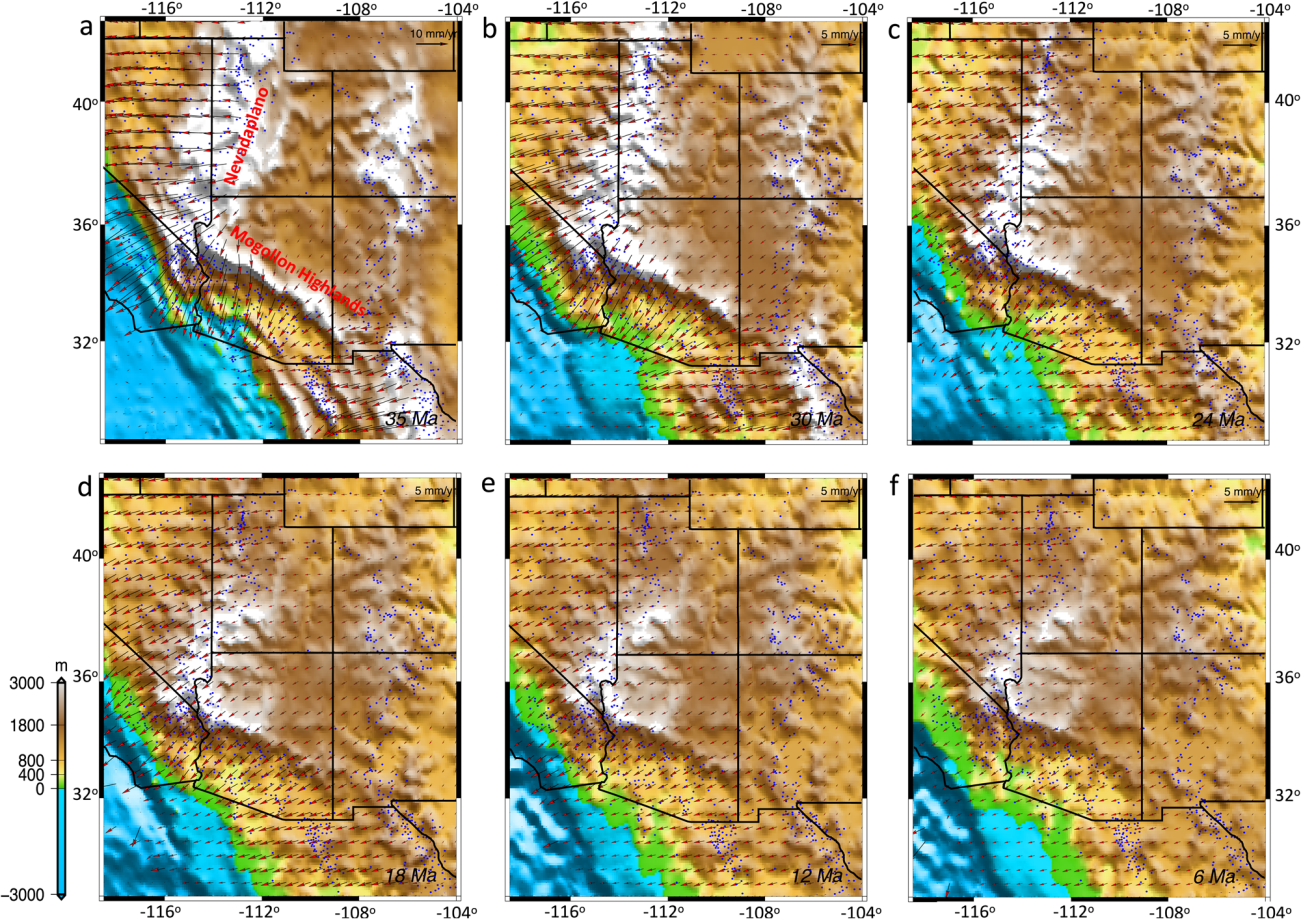
Kinematic evolution of the upper crust of the North American lithosphere. **a**–**f** Red arrows represent displacement rates of the upper crust at 35, 30, 24, 18, 12, and 6 Ma, respectively. Blue spots are the location of normal fault offset observations from McQuarrie and Wernicke^20^ and Bahadori et al.^9^. The map images were created by authors using: www.soest.hawaii.edu/gmt/ and www.paraview.org/.

**Fig. 9:**
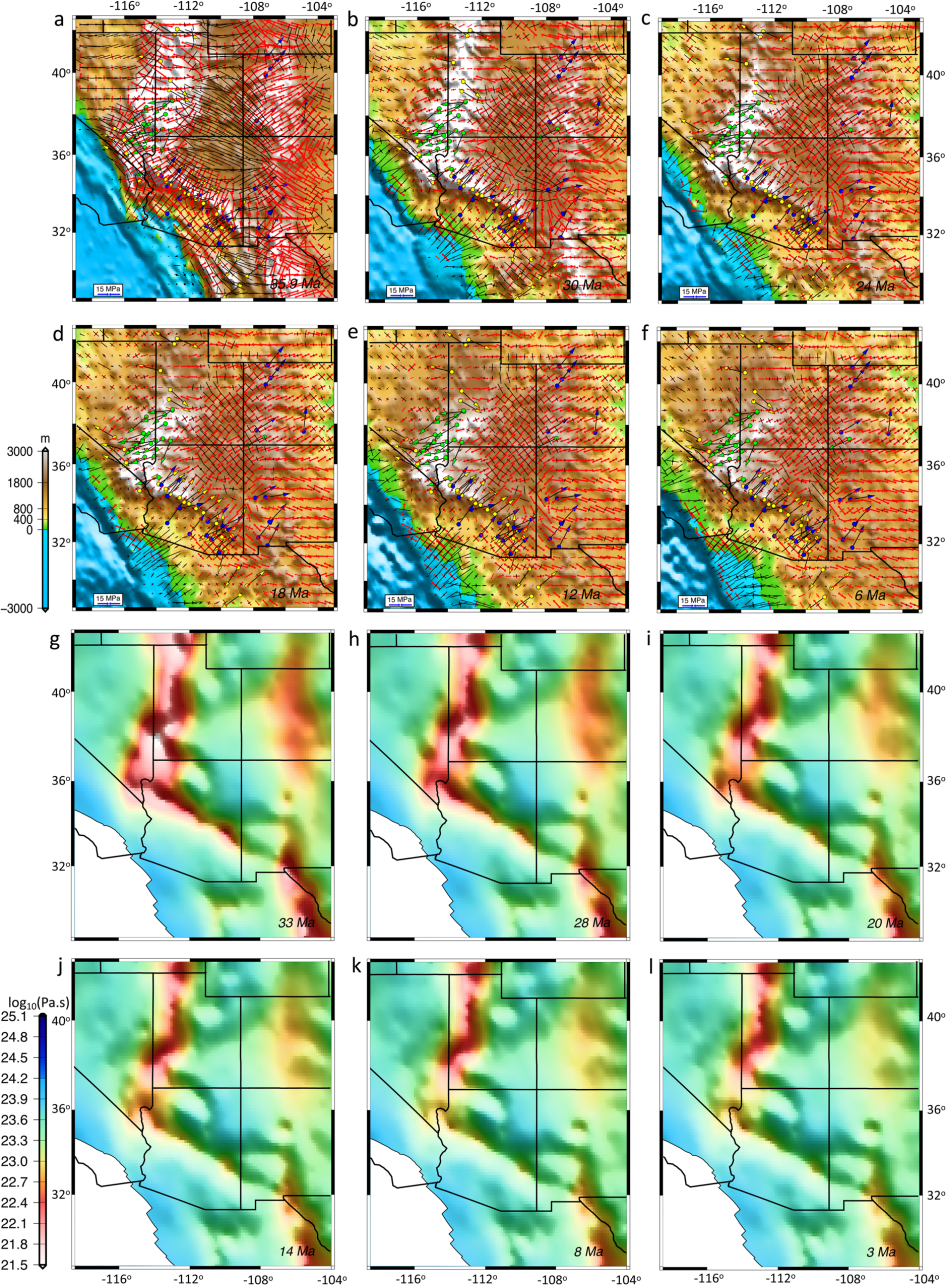
The evolution of principal axes of deviatoric stresses for the upper crust and effective viscosity within the lithosphere in southwestern North America. **a**–**f** Variation of the principal axes of horizontal deviatoric stresses for the upper crust at 35.9, 30, 24, 18, 12, and 6 Ma, respectively. Black arrows represent compressional and red arrows represent tensional stresses. The color-coded circles and vectors represent the stretching direction of metamorphic core complexes shown in yellow and Miocene faults and dykes trend shown in green and blue. **g**–**l** Depth averaged effective viscosity of the North American lithosphere, from surface to the base of the lithosphere, at 33, 28, 20, 14, 8, and 3 Ma, respectively. The map images were created by authors using: www.soest.hawaii.edu/gmt/ and www.paraview.org/.

The constraint of sub-lithosphere tractions associated with our mantle convection model (Fig. 4) along the base of the NA lithosphere results in an eastward basal flow (Fig. 5), which causes shearing and advection of material into the interior of the NA lithosphere through a zone of high finite strain within the mantle asthenosphere (Figs. 6 and 7). In our simulations, conductive heating weakens the lithosphere, and the thermal erosion and basal shearing of the mantle lithosphere beneath the NA Plate progress within areas of high topographic collapse (Figs. 6 and 7). Our models show the presence of lower crustal flow during the crustal stretching process that widens the zones of paleo-highland in Nevada and Arizona (Figs. 8 and 9). In our simulations, the free-boundary collapse of an over-thickened crust^6^ predicts differential motion between upper brittle and weak lower crust. Such lower crustal flow is limited to the regions containing crustal welts (Figs. 6 and 7). Therefore, while the exhumed ductile middle-lower crust in MCCs of SWNA imply the presence of lower crustal flow during the stretching process, our model shows that this lower crustal flow should have been limited in scale. Owing to a weak lower and middle crust, the eastward basal shear in the mantle asthenosphere has little influence on the horizontal extension within the BRP over time, which is driven by the high GPE of the highlands (Figs. 6 and 7). The thermal erosion of the mantle lithosphere and overlying crustal extension results in the thinning of the ~60 km crustal root of the Nevadaplano and the Mogollon Highlands through a free-boundary collapse of an over-thickened crust, which eventually results in a ~30 km crust within the BRP at the present-day in our model (Fig. 6). The rapidly deforming mantle below the BRP in comparison to the little deformed mantle below the Colorado Plateau (CP) results in a shallow lithosphere (70–80 km) underneath the BRP, a thicker lithosphere (100–110 km) underneath the CP, and a more than 110 km thick lithosphere underneath the Great Plains (Figs. 6 and 7).

Our starting paleo-elevation model for the late Eocene includes a substantial belt of continuous high topography in Nevada and western Utah (Nevadaplano) as well as Arizona and northwestern Mexico (Mogollon Highlands) (Fig. 8a). The highlands in Arizona and northwestern Mexico undergo progressive collapse from 33 to 20 Ma, and the highlands in Nevada and western Utah undergo collapse since the late Eocene (Fig. 8). By quantifying the magnitudes of deviatoric stresses associated with lithospheric body forces and mantle convection over time, we test the relative contributions of these two driving mechanisms on the extension and topographic collapse that led to the present-day BRP. A remarkable feature is that, owing to the presence of paleo-highlands and the associated high GPE, deviatoric stresses are dramatically increased just prior to extensional collapse of topography (Fig. 9a, Supplementary Movie 4). The consequence of extensional deviatoric stresses associated with highlands surrounding the CP is a contractional stress field within the CP at the late Eocene (Fig. 9a). This stress regime should have been in place since the Sevier and Laramide orogeny and during the flat subduction of the Farallon Slab underneath the NA plate and before the weakening of the lithosphere following the slab rollback. During the initial phases of topographic collapse, extension is limited to a zone along the highest paleo-topography, with stretching in northwest-southeast (central-northern Nevada) and northeast-southwest (Arizona and northwest Mexico) directions (Figs. 8 and 9). Through time, as the paleo-highlands collapse, the GPE magnitude decreases and magnitude of extensional deviatoric stresses drop by ~90% at around 10 Ma. This results in a more uniform distribution of GPE within the BRP following 10 Ma (Fig. 9). Our time-dependent models show that extensional collapse of highlands through time is facilitated by low effective viscosities of the lithosphere (Fig. 9). This reduction in effective viscosity results from higher temperatures beneath the highlands and partial melting of the deep crust in our models (Fig. 5, Supplementary Figs. 3 and 4), and is consistent with the felsic magmatism in the area for this time interval^9,49^ (Fig. 1a).

During the Oligocene to early Miocene (33–20 Ma), the lithosphere within the Nevadaplano region gradually undergoes an increase in effective viscosity, followed by a shift to east-west extension within the northern-central Nevadaplano region (Fig. 9). This increase in effective viscosity is associated with a reduction in partial melting of the deep crust, which is in agreement with the timing that felsic magmatism migrates to the west following slab rollback^9,49^ (Fig. 1a, Supplementary Figs. 3 and 4). During the middle to late Miocene (20–6 Ma) and following the collapse of the Mogollon Highlands, there is crustal thinning and topographic collapse of the southern Nevadaplano (Fig. 8). In contrast to the Nevadaplano region, Arizona undergoes an effective viscosity increase during 20–10 Ma which is associated with partial melt reduction of the deep crust (Fig. 9, Supplementary Fig. 4). Topographic collapse and reduction of GPE magnitude, together with an increase in effective viscosity of the lithosphere result in smaller displacement rates and extension within the BRP post-10 Ma (Fig. 8).

Our model shows that thermal erosion of the mantle lithosphere and lower crustal flow at the western edge of the CP tilts the plateau to the east and northeast by 20 Ma. The timing of CP edge surface uplift from 25 to 15 Ma in our simulations is consistent with incision observations^35^ during Neogene surface uplift for the CP edge with respect to the BRP. As surface uplift continues to the center and eastern part of the CP by 15 Ma, the model shows the doming of the central part of the CP (Fig. 8). Late-stage lithospheric strength distribution in our models (Fig. 6f) is consistent with present-day effective elastic thickness distribution for SWNA^50^. Moreover, following 36 Myr of finite strain, our simulated final Moho boundary and lithosphere-asthenosphere boundary (LAB) structures are consistent with interpretations of seismic profiles across the region^51^ (Fig. 6f).

### Climate and surface processes influences on landscape evolution

**Fig. 10:**
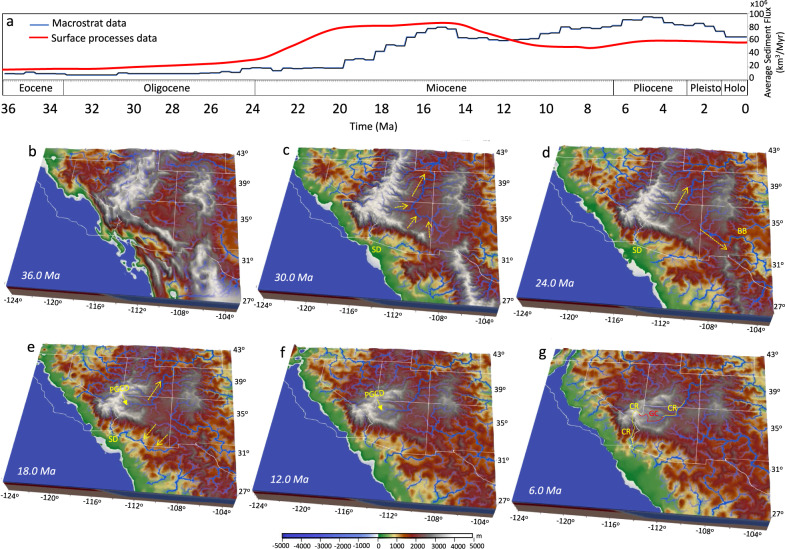
Interaction and interconnection between tectonics, climate, and surface processes. **a** Dark blue line represents the average sediment flux within the Basin and Range Province based on the palinspastically restored history of sediments from Macrostrat since the late Eocene. Red line represents simulated average sediment flux within the Basin and Range Province based on surface processes simulations from the late Eocene to present. **b**–**g** Simulated paleo-elevation and drainage in southwestern North America at 36, 30, 24, 18, 12, and 6 Ma, respectively. Panel ‘c’ shows north and northeast drainage onto the Colorado Plateau from the Nevadaplano and Mogollon Highlands. Panel ‘d’ shows a drainage reversal to the south. Panel ‘e’ shows drainage out of the Colorado Plateau to the southwest. SD Sespe Delta, PGCD Paleo Grand Canyon Drainage, GC Grand Canyon, CR Colorado River, BB Baca Basin. The map images were created by authors using: www.soest.hawaii.edu/gmt/ and www.paraview.org/.

The modeling of paleo-climate and surface processes predict three phases of shifting drainage patterns that significantly affected sediment supply within the foreland and hinterland of the Sevier thrust belt and that provides general agreement with palinspastically restored history of sediment packages (Fig. 10a). Below, we describe major phases of drainage evolution that result from our model.

In the late Eocene, rivers entered the CP from the Mogollon Highlands in the south and the Nevadaplano in the west (Fig. 10, Supplementary Movie 5). These highlands directed flow to the north or northeast along paleo-channels^52^, in accord with gravel deposits^53–56^. During the late Eocene to 28 Ma, the internal drainage continued to flow northerly from the Mogollon Highlands and easterly off the Nevadaplano with no obvious outflow from the CP (Fig. 10a, b). As such, the recorded sediment flux within the Great Basin (GB) and southern BRP (Macrostrat data^48^) is low prior to 24 Ma (Fig. 10a). Our model demonstrates that this low sediment flux is associated with paleo-rivers that flowed off the Nevadaplano and the Mogollon Highlands prior to and during their extensional collapse (Supplementary Fig. 5).

Following the collapse of the Mogollon Highlands, our tectonic forcings predict a drainage reversal in Arizona south of the CP at ~24–18 Ma (Fig. 10d, e). The surface uplift of the central portion of the CP, together with topographic collapse of the eastern side of the CP between 20–15 Ma diverts drainages away from the center of the CP. The significant drainages exiting the southern CP deposited substantial volumes of sediment in parts of the southern BRP and at the river mouth (coastline, or Sespe Delta^57^) (Fig. 10e). This result is generally consistent with the palinspastically restored history of sediment packages from Macrostrat^48^, which show a dramatic increase in sediment flux within the southern BRP post-22 Ma (Fig. 10a and Supplementary Fig. 5).

Northeast drainage off highlands along the southwest margin of the CP persist in the model up to 6 Ma (Fig. 10e–g). A paleo Grand Canyon drainage (PGCD), that developed between the late Eocene to 10 Ma from river systems flowing east out of the paleo-highlands of the southern Nevadaplano and onto the CP (Fig. 10b–f), could have later been captured by the ancestral Colorado River during later drainage reversal and southwest directed outflow into the Colorado River Extensional Corridor (CREC) at 6 Ma.

## Discussion

Our geodynamic model pulls all the key elements together to quantify the sources and primary driving factors for deformational features in SWNA since the late Eocene. Based on our results thermal erosion and east-directed basal shearing of the weak and hydrated NA lithosphere together with the thermal perturbation and conductive heating within the NA lithosphere can explain the onset of the Cenozoic magmatism onto the BRP and CP as well as weakening of the mantle lithosphere and lower crust. This weakening led to the onset of the extensional collapse of the paleo-highlands (Figs. 6–10). Our lithosphere model demonstrates a strong correlation between high topography, crustal root, partial melting of the deep crust, lower crustal flow, asthenosphere influx, and weakening of the lithosphere with reconstructed stretching history^10^, evolution of paleo-volcanism^10,49^, and key geologic stretch directions^21^.

For example, the magnitude and direction of the eastward flow at the base of the lithosphere, in our models, agree with shallow asthenosphere flow fields in recent models of the evolution of mantle thermal structure and flow field below SWNA post mid-Cenozoic^18^. The magnitudes of eastward mantle flow just below the lithosphere in the models of Zhou et al.^18^ and Zhou and Liu^58^ are around 2–4 cm/yr and these basal velocities are distributed eastward horizontally over distances of hundreds of kilometers beyond the trench position. This shallow eastward flow of mantle asthenosphere relative to an upper lithosphere that is undergoing BRP extension and transform margin-associated shear (Fig. 6) agrees with results inferred from shear-wave splitting observations^59,60^. Therefore, our 4-D simulations of the lithosphere evolution in SWNA includes this important basal velocity boundary condition (Fig. 5) that was a likely consequence of the evolving subduction process following the slab rollback history. The thermal erosion and basal shearing of the mantle lithosphere and reduction in effective viscosities alter the lower 5–10 km of mantle below the CP to behave like asthenosphere (Fig. 7). This is consistent with findings of Li et al.^61^ who argued for thinning of the CP lithosphere by dissociation based on higher water content in mantle xenoliths from beneath the CP in comparison with a typical continental lithosphere. This lithosphere thinning under the CP, that our simulation shows, is also consistent with findings of Riter and Smith^62^ and West et al.^63^ who argued for a maximum of ~5–010 km of Cenozoic lithosphere thinning based on xenoliths and tomography data, respectively, and that thick lithosphere likely no longer exists beneath the outermost edge of the plateau^64^.

Our simulations demonstrate that collapse of the highlands in SWNA is facilitated by weakening of the lower crust. This collapse involves extension of a thick crustal welt with a partially melted lower crust, leading to the thinning of the crust there (Figs. 6 and 7). There is isothermal decompression of the hot deep crust resulting in decompression melting and formation of a flat Moho, which could potentially facilitate the development of MCCs^10^. In our experiments, we have determined that a reduction in density and viscosity owing to the presence of melt and a partially melted lower crust for paleo-highlands, in comparison to a starting lower crust without any melt, increases the mobility of the deep crust, which flows in response to lateral pressure gradients. The melt fraction produces some upward and lateral advection of heat and material that contributes to the weakening of the crust and causes the topographic collapse through extension of the upper crust as well as thinning of the crust by flow of the lower crust (Figs. 5a and 6–8). Our experiments show that such a mechanism can explain the transition from a ~60 km crustal welt to a ~30 km crust within the BRP following slab rollback. The comparison between the magnitude of the surface velocities generated from our 4-D thermo-mechanical model within the BRP (Fig. 8) with horizontal surface motions of Bahadori and Holt^10^, determined based on a compilation of fault displacements from the model of McQuarrie and Wernicke^20^, shows a good agreement between our model output velocities at the surface and those determined by geological observations.

The comparison of our model outputs for stress tensors and their associated principal axes of horizontal tensional deviatoric stresses within the upper crust matches the Miocene fault and dike trends^65^ and MCC lineation directions^21^, but with the added advantage that the calculations involve a full 4-D thermo-mechanical response of the crust and upper mantle (Fig. 9). In the early stages of collapse of the Nevadaplano, during the late Eocene to early Oligocene, there is northwest-southeast extension in Nevada that is in agreement with vergence of Cordilleran MCCs^21^ and orientations of stretch there. This northwest-southeast extension is at an angle of ~45° to the contractional stresses and is limited to highest GPE zones in eastern Nevada and western Utah (Fig. 9a). This feature of the geodynamic model is an important analog to the present-day setting in the Altiplano of South America, which shows extension directions at highest elevations at an acute angle of ~45° to the contractional stresses^66^. Our model shows that high GPE of the mountain chain in Arizona (Mogollon Highlands) could produce tensional deviatoric stresses that were a fundamental driving mechanism for extension and topographic collapse until 20 Ma, when GPE magnitude drops significantly. Garnet–pyroxenite xenoliths^51^ and geochemical evidence^67^ support the presence of a thick crust and high elevation (high GPE) in Arizona at least until 24 Ma. A naturally constrained stress profile through the middle crust (25 km depth) for the footwall of the Whipple Mountains MCC^68^ indicates a deviatoric stress range of 10–30 MPa during the Miocene extension in Arizona, in agreement with our model deviatoric stress values of >10 MPa for the upper crust and brittle–ductile transition (Fig. 9). The northeast-southwest tensional oriented deviatoric stresses within Arizona (Mogollon Highlands) during topographic collapse is in good agreement with key geologic stretch directions obtained from vergence of Cordilleran MCCs^21^, Miocene fault trends^69^, and Miocene dyke trends^65^ (Fig. 9). The east-northeast to west-southwest extensional orientation of tensional deviatoric stresses during the middle to late Miocene within the western Arizona and southern Nevada agrees with the stretch direction of MCCs and Miocene fault trends (Fig. 9).

Our results demonstrate that, in addition to high GPE magnitudes in Nevada and Arizona during topographic collapse and MCC development, the weakening of the lithosphere is required to explain the initiation of high extensional strain rates^10^ there (Fig. 9). Such weakening in our model is caused by partial melting of the deep crust associated with an increase in upper mantle temperature following slab rollback (Supplementary Figs. 3 and 4). Felsic and mafic volcanism within the belt of extensional collapse from southeast to northwest Arizona and from northeast to southwest Nevada^9,49^, during the late Eocene to 15 Ma (Fig. 1a), support partial melting and reduction of effective viscosity of the lithosphere.

The agreement between the direction of principal axes of tensional deviatoric stresses in our time-dependent geodynamic model and the locations of key geologic stretch directions obtained from vergence of Cordilleran MCCs as well as Miocene fault and dyke trends show that smaller-scale convection (slab rollback) is unlikely to have significant impact on the stress field within the NA lithosphere through its introduction of tractions to the base of the lithosphere. Moreover, the temporal and spatial correlation between magmatism and lowering of lithosphere effective viscosity in our model suggests that slab rollback likely played a critical role in lithosphere weakening through the introduction of heat, melts, and fluids consistent with findings of Bahadori and Holt^10^ (Figs. 1 and 9).

The timing and mechanism of extensional collapse and surface uplift history within the BRP and CP regions produce external forcing conditions for our surface processes simulations. Our performed reconstruction the modeling for paleo-streams and sediment distribution in space and time indicates that such external forcings can provide a close fit between our model outputs for sediment distributions and volumes and geological observations, based on records of depositional sequences^48^ (Fig. 10a). The three predicted phases of drainage evolution that our time-dependent simulations show appear to match, in time and space, the major sedimentological and drainage features from the late Eocene to present-day (Fig. 10b–g). The period of internal drainage onto the CP during the late Eocene to early Oligocene overlaps in time with the deposition of the Rim Gravels^52–56,70^, the Chuska Sandstone Formation deposition in northeastern Arizona and northwestern New Mexico during the final episode of Paleogene aggradation on the central and southern CP^52,71^, including outflow from the eastern CP to the Baca Basin in central New Mexico (Fig. 10d). A long-lived northward draining paleo-river system across the entirety of the CP, along with a drainage divide on the west and south of the CP, is consistent with fish fauna^3^ that suggests CP species in the Miocene Lake Bidahochi formation hold similarities with those originating from the Snake River system far to the north.

During the late Eocene to late Oligocene when our model shows northeast drainage on top of the CP, our model also shows a drainage, originated on the southwest flank of the Mogollon Highlands, that flowed southwest into the Pacific. This deposition result from our model is consistent with paleo-geographic reconstruction of Howard^57^ who argued for a paleo-river system sourced in the Mogollon Highlands that flowed into what is now known as the Los Angeles trough region, called the Sespe Formation conglomerate (Fig. 10b, c). Our model shows that a second phase of deposition into the Sespe delta region occurs following the collapse of the Mogollon Highlands and southern Nevadaplano during 20 to 10 Ma, where paleo-streams exit the southern CP in Arizona flowing southwest (Fig. 10e).

Besides the crustal extension, topographic collapse, and landscape deformation within the BRP, the model also provides potential implications on other observations, such as surface uplift within the CP, but a detailed discussion is beyond the scope of this paper.

We also want to emphasize some model caveats. First, while geological observation^72^ suggests that extension and subsidence in the CREC led to deposition of the Muddy Creek Formation at ~13 Ma along the mouth of the Grand Canyon, the model does not produce the broader opening of the CREC following 15 Ma. This is perhaps because our model does not simulate formation of a transform boundary between the Pacific and NA Plates following 15 Ma, promoting the transtensional deformation to the southwest margin of the CP^10^. Insufficient crustal extension along the southwestern margin of the CP results in the persistence of a northeast directed gradient there. We speculate that sufficient extension within the CREC post-15 Ma would result in a drainage reversal of the paleo-Colorado river and eventual outflow through the older drainage cut by northeast flow from the highlands (PGCD; Fig. 10g) into the CREC. This could potentially cause formation of the modern Colorado River that would connect several old pre-existing segments of the Grand Canyon consistent with findings of Karlstrom et al.^34^ (Fig. 10g). Second, while our model shows an east-northeast drainage onto the CP prior to Miocene extensional collapse of highlands, there are, however, studies that argue for a pre-late Eocene California River outflow to the west-southwest through the southwest corner of the CP that carved the Grand Canyon during early Eocene (e.g., Wernicke^37^). It is important to mention here that our model at the late Eocene has a very large topography that acts as a major barrier to any drainage exiting the southern CP prior to 20 Ma. In addition to the results from the restoration of extensional history^9,31^, recent isotope analyses and geochemical evidence^67^ support the presence of an orogenic highland with a ~60 km thick crust and an associated paleo-elevation of ~3 km in SWNA along the edge of the CP during the Laramide. Therefore, an older Grand Canyon with southwest flow would need to involve a major drainage cut through the continuous chain of high topography in the southernmost part of the Nevadaplano, or northwesternmost part of Mogollon Highlands (Kingman Arch). However, regional topographic gradients do not favor a southwest flow prior to 15 Ma, and possibly not prior to 10 Ma, owing to the lower elevation of the CP with respect to the highlands to the south and southwest. Furthermore, Eocene outflow to the Pacific is not consistent with fish fauna^3^ within the CP province, which supports only outflow to the Atlantic during the late Eocene to Oligocene and argues for a persistent barrier between the CP and the Pacific Ocean. Third, while overall there is a good agreement between the pattern and magnitude of sediment flux within the BRP from the Macrostrat data and our simulated results (Fig. 10a), our simulation indicates a sharp increase of sediment flux at 24 Ma and a gradual decrease of sediment flux a little bit after 14 Ma, which is not observed in the reconstructed sediment history from the Macrostrat data. We interpret these misfits to be associated with uncertainties embedded in our reconstructed paleo-climate model together with the nature of the one-way coupling between the paleo-climate and the geodynamic and landscape evolution.

Together, our tectonic and landscape evolution modeling helps to resolve long-standing problems linking mantle convection, lithosphere dynamics, and extensional collapse of highlands to the evolution of major river systems, with implications for the history of the paleo-Colorado River and incision of the Grand Canyon. We argue that high GPE associated with the presence of paleo-highlands, supported by a thick crustal root, is required to explain stress magnitudes and strain orientations along the belt of MCCs in SWNA. As such, while slab rollback was likely critical in the strength evolution of the lithosphere, body forces were the major factors dictating the style of strain history and topographic collapse from the late Eocene to ~10 Ma. This major result confirms the importance of paleo-topography and paleo-crustal structure in driving the early extensional history and demonstrates that our quantified lithospheric stresses can constrain the forces that derive the style of lithospheric deformation in SWNA. Thus, tractions associated with smaller-scale convection, such as slab rollback before, during, and after the collapse of the paleo-highlands, played a much lesser role in their influence on the stress field than do the GPE gradients, which are modeled in this study. The agreement between predictions from our coupled tectonics, climate, and surface processes models and superficial geological record for sediment distribution and paleo-canyons indicates the importance of reconstruction of erosional and depositional histories and paleo-drainage systems within the context of the evolving dynamic system. We propose that, this type of approach offers an opportunity to refine our understanding of landscape evolution on Earth, and that the sedimentary record is an important constraint on coupled tectonic-climate-landscape models.

## Methods

### Numerical experiment setup, boundary conditions, and solutions for lithosphere thermo-mechanical simulation

We develop 4-D realistic coupled thermal and mechanical tectonic and geodynamic models of the lithosphere using open-source Underworld Geodynamics (UWGeodynamics) code^73–76^. UWGeodynamics accounts for conservation of mass, energy, and momentum, and utilizes a Lagrangian integration Particle-In-Cell Finite-Element-Method approach (tracking particles embedded in the deforming material relative to the mesh) for the solutions to incompressible Stokes flow type configurations and heat transport equations. Fundamental UWGeodynamics equations for conservation of mass, energy, and momentum include:

1 $$\nabla \cdot u=0,$$

2 $$\rho {C}_{p}\left(\frac{\delta T}{\delta t}+u\cdot \nabla T\right)=\nabla \cdot \left(k\nabla T\right)+Q,$$

3 $$\nabla \cdot (\eta \nabla u)-\nabla p=-\rho g,$$

where *u* is the velocity, *T* is the temperature, *t* is time, ${C}_{p}$ is the specific heat capacity, *ρ* is the density, *k* is the thermal conductivity, *Q* is an additional heat source for the energy equation, ∇*u* is the velocity gradient, ∇*p* is the pressure gradient, *η* is the viscosity, *ρg* is the driving force, and ∇ ∙ (*η*∇*u*) is the stress gradient.

In our simulations, we incorporate additional geodynamic terms like radiogenic heating (*H*) and the thermal aspects of partial melting (*F*) into Eq. (2) for conservation of energy as

4 $$H=\frac{{R}_{h}}{\rho {C}_{p}},$$

5 $$F=-1\times \left(\frac{{L}_{f}}{{C}_{p}}\right)\left(\frac{{\partial M}_{f}}{\partial t}\right),$$

where ${R}_{h}$ is the rate of radiogenic heat production, ${L}_{f}$ is the latent heat of fusion which represents the amount of energy consumed during a phase change from solid to liquid, and ${M}_{f}$ is the melt fraction. *H* specifies the magnitude of energy that is added into the system. Therefore, in our simulations, the rate of change of temperature as a function of heat diffusion (${\alpha \nabla }^{2}T$), heat advection (*u*∙∇*T*), radiogenic heat production (*H*), and heat changes associated with partial melting processes (*FT*) is expressed as

6 $$\frac{\partial T}{\partial t}={\alpha \nabla }^{2}T-u\cdot \nabla T+H+{FT},$$

where *α* is the thermal diffusivity $(\alpha =\frac{k}{\rho {C}_{p}})$. Equation (6) ensures conservation of energy and allows the coupling of densities and viscosities to temperature.

The modular structure of UWGeodynamics allows for implementation of highly complex mechanical models. With this code, we describe the initial and boundary conditions, the material properties, rheological laws, and specialized processing of the numerical solution to relevant geophysical outputs. Our 4-D thermo-mechanical model consists of a 3-D Cartesian domain that includes a longitudinal range of 124°W to 103°W (~2100 km), a latitudinal range of 27°N to 44°N (~1700 km), and a maximum lithosphere depth of 200 km, which is solved by 43 × 35 × 50 = 75250 Eulerian nodes and 42 × 34 × 49 = 69972 finite-element cells on a finite-element mesh. We use the model of Bahadori and Holt^10^ to generate a starting 3-D crustal and upper mantle structure for UWGeodynamics with a resolution of 20 km × 20 km (approximately ${0.2}^{^\circ }$ × ${0.2}^{^\circ }$). The model also includes a lithosphere thickness of 120 km and an asthenosphere thickness of 80 km. We then assign specific properties listed in Supplementary Table 1 to each material included in our model.

The free-slip boundary condition has been applied to the left, right, front, and back of the model, and a stress boundary condition achieved from mantle convection simulation has been applied to the base of the model at 200 km depth. A “sticky-air” layer is defined on top of the elevation to simulate a free surface. The advantage of introducing a “sticky-air” layer into our simulation is that it allows topography to evolve naturally. The real value of viscosity for the air (1.81 × 10^−5^ Pa s) would make our model unstable so that in order to retain isostatic balance, a very small time step should be employed to avoid the “sloshing instability” or the “drunken sailor effect”^77^, which makes the simulations computationally very expensive. However, the high viscosity of 1.0 × 10^19^ Pa s that we use for the “sticky-air” is still low enough that does not affect the geodynamics of our simulations. This allows the interface of air/crust to behave similar to a natural free surface^78^. As such, since there is not an interface with large viscosity contrast, the simulations are a lot more stable.

We also solve for the thermal evolution of the model through time. The simulation is initiated with a temperature field that is derived from solving a transient coupled thermo-mechanical model wherein the velocity boundary conditions at the left, right, front, and back of the model are set to 0 cm/yr. The initial temperature field for the model is defined based on a linear initial geotherm^79^ with a thermal boundary condition at the top of the model that is the absolute temperatures of 273 K, a laterally varying temperature field at the base of the model (200 km depth) achieved by adding 400 K (5 K/km) to the temperature model of Bahadori and Holt^10^, and a constant basal heat flow of 200 mW m^−2^ together with zero heat flow across the lateral sides of the model. To simulate the effect of slab rollback and temperature variations at the base of the NA lithosphere, the starting temperature boundary condition at the base of the model is updated using a time-dependent thermal boundary condition that is defined based on absolute temperatures at the base of the model that account for temperature changes from the model of Bahadori and Holt^10^. The model of Bahadori and Holt^10^ was based on the reconstructed (in position) magmatism as a proxy for centers of temperature perturbations over time^9^ and these patterns of volcanic centers are assumed to reflect slab rollback history.

In addition to solving the thermal evolution of the model, we also solve the equilibrium equations for viscous–plastic flow in three dimensions. Therefore, in our simulations material deformation is expressed based on non-Newtonian visco-plastic rheologies with viscosity dependent on temperature, pressure, and strain rate. The implementation of lithospheric deformation in terms of a visco-plastic rheology is incorporated by decomposing the deviatoric strain rate into viscous and plastic components that are solved sequentially. The flow is computed through dislocation creep (*n* > 1 and *G* = 0, where *n* is the power-law stress exponent and G is the grain size exponent)^80,81^ for the viscous component, which can be expressed using the following equation:

7 $${\eta }_{{{{{\rm{eff}}}}}}^{{{{{\rm{viscreep}}}}}}(T,P,\dot{\epsilon })=f{A}^{\frac{-1}{n}}{\dot{\epsilon }}^{\frac{(1-n)}{n}}{{\exp }}\left(\frac{E+{PV}}{{nRT}}\right),$$

where *A* is the pre-exponential factor that is not sensitive to thermo-chemical conditions, *n* is the stress exponent and is a non-dimensional constant, $\dot{\epsilon }$ is the strain rate, *E* is the activation energy, *P* is the pressure, *V* is the activation volume, *R* is the gas constant, *T* is the temperature, and *f* is the scaling factor chosen to represent materials that are viscously weaker or stronger than the reference flow law.

For the plastic component of the flow (frictional-plastic yielding), failure is determined using a pressure-dependent Drucker–Prager yield criterion, which is equivalent to the Mohr–Coulomb yield surface for incompressible deformation^81^:

8 $${\sigma }_{{{{{\rm{yield}}}}}}={({J}_{2}^{\prime})}^{1/2}={Ap}+B=\frac{6{\sin }{{\emptyset }}p}{\sqrt{3}(3-{\sin }{{\emptyset }})}+\frac{6C\;{\cos }{{\emptyset }}}{\sqrt{3}(3-{\sin }{{\emptyset }})},$$

where ${J}_{2}^{\prime}$= $\frac{1}{2}$ ${\sigma }_{{ij}}^{\prime}{\sigma }_{{ij}}^{\prime}$ is the second invariant of the deviatoric stress tensor, ${\sigma }_{{ij}}^{\prime}$ is the deviatoric stress tensor, *C* is the cohesion, ∅ is the internal angle of friction, and *p* is the pressure. In the crust and mantle, frictional sliding is modeled via Mohr–Coulomb criterion with a starting cohesion of 10 MPa and a coefficient of friction of 0.1^82^. In our simulations, the brittle properties of materials change as a result of a local strain accumulation so that both cohesion and friction coefficient decrease linearly with strain. For all materials in our simulations, the yield stress linearly drops to maximum of 20% of its initial value (or 2 MPa) when the accumulated strain reaches 0.5. Hence, during frictional strain softening, the friction coefficient (µ) reduces linearly from 0.1 to 0.01 for brittle strain between 0.0 and 0.5. For strains equal to 0.0 ($\epsilon = < \, {\epsilon }_{1}$), it remains constant at 0.1 (${\mu =\mu }_{{{{\bf{0}}}}}$), and for strains larger than 0.5 ($\epsilon \, > \, {\epsilon }_{2}$), it remains constant at 0.01 ($\mu ={\mu }_{{{{\rm{\infty }}}}}$). Hence, strain accumulation causes weakening of the material mechanically so that the friction coefficient and cohesion are softened, and once fully weakened, they maintain a steady state (Supplementary Table 2). Therefore, under lower pressure and high stress conditions, when differential stresses reach the yield stress, the material fails, and deformation is modeled by an effective viscosity as

9 $${\eta }_{{{{{\rm{eff}}}}}}^{{{{{\rm{plastic}}}}}}=\frac{{\sigma }_{{{{{\rm{yield}}}}}}(p,\dot{\epsilon },\epsilon )}{2\dot{\epsilon }},$$

where $\dot{\epsilon }={(\frac{1}{2}{\dot{\epsilon }}_{{ij}}{\dot{\epsilon }}_{{ij}})}^{1/2}$ is the second invariant of the strain rate tensor. As strain is accumulated, yielding rheologies linearly interpolate between their original values (e.g., cohesion, friction coefficient) to their damaged values (Supplementary Table 2). As pressure increases, the deformation mechanism of a material changes from plastic to viscous (ductile). As such, in our simulations when the state of stress is below the frictional-plastic yield, the flow is viscous and is specified by temperature-dependent nonlinear power-law rheologies based on laboratory measurements for dislocation creep on wet quartz^83^ for the upper crust, wet diorite^84^ for middle crust, dry Maryland diabase^83^ for lower crust, strong dry Maryland diabase^83^ for oceanic crust as well as lower crust of the Colorado Plateau, wet olivine^83^ for a hydrated mantle lithosphere consistent with the study by Bahadori and Holt^10^ for southwestern North America, and dry olivine^80^ for mantle asthenosphere (Supplementary Table 3). The viscous creep and plastic yielding are combined by assuming that they act in parallel as independent processes. Hence, the effective visco-plastic rheology is calculated as

10 $${\eta }_{{{{{\rm{eff}}}}}}^{{{{{\rm{visco}}}}}{\mbox{-}}{{{{\rm{plastic}}}}}}={{{\rm{min }}}}\,({\eta }_{{{{{\rm{eff}}}}}}^{{{{{\rm{viscreep}}}}}},{\eta }_{{{{{\rm{eff}}}}}}^{{{{{\rm{plastic}}}}}}).$$

In order to mitigate an untractable stiffness matrix because of its poor conditioning that can be associated with very small strain rates and predicted high viscosities in regions where the fluid behavior is rigid, or very large strain rates and predicted small viscosities in regions where the deformation is localized, we define two viscosity thresholds of ${\eta }_{{\min }}=$1e^18^ and ${\eta }_{{\max }}=$1e^24^. Hence, ${\eta }_{{\min }}\le {\eta }_{{eff}}\le {\eta }_{{\max }}$

In our simulations, the mechanical effect associated with partial melting of the lower crust within the Basin and Range Province is determined by reduction of the viscosity of the lower crust within a melt range of 0.15 to 0.3. Melting is applied to an existing viscous rheology, and is calculated as

11 $${M}_{{{{{\rm{int}}}}}}=1+\left(\frac{{M}_{f}-{L}_{f}}{{L}_{f}-{U}_{f}}\right).$$

12 $${\eta }_{m}=\eta \times ({1+M}_{{{{{\rm{int}}}}}}+{\eta }_{f}\times (1-{M}_{{{{{\rm{int}}}}}})),$$

where ${\eta }_{m}$ is the updated viscosity after material melts, *η* is the viscous rheology, and ${M}_{{{{{\rm{int}}}}}}$ is a normalized linear interpolation of the percentage of the melt fraction (${M}_{f}$) between the upper (${U}_{f}=$30%) and lower (${L}_{f}$ = 15%) limit of the melt fraction range, and ${\eta }_{f}$ is the melt viscous softening factor that lower crust material undergoes once melted (Supplementary Table 4). In our experiments, when the melt fraction increases from 15% to 30%^8,85^, the viscosity decreases by 2 orders of magnitude. Therefore, when the melt fraction is 15%, the viscosity of the melted crust is that of the non-melted surroundings, and when the melt fraction is 30%, the viscosity of the melted crust is 100 times lower than in the surrounding material. It is important to mention that segregation of the melt from the host rock does not happen in our simulations and melt phase remains in its source, which is consistent with observations of migmatite-cored metamorphic core complexes in which a relatively small volume of leucogranite is extracted from the partial melt layer^86,87^.

In our simulations, the initial relation between temperature and density is expressed as

13 $$\rho (T)={\rho }_{0}[1-\beta \left(T-{T}_{0}\right)],$$

where ${\rho }_{0}$ is the reference density, *β* is the coefficient of thermal expansion (3 × 10^−5^ k^−1^), *T* is the temperature, and ${T}_{0}$ is the reference temperature. The melt fraction (${M}_{f}$) in our simulations is a function of the super-solidus dimensionless temperature^88^ and is calculated as

14 $${T}_{{ss}}=\frac{(T-({{T}_{s}+{T}_{l}})\times 0.5)}{({T}_{l}-{T}_{s})},$$

15 $${M}_{f}=0.5+{T}_{{ss}}+({T}_{{ss}}^{2}-0.25)\,\times(0.4256+2.988\times{T}_{{ss}}),$$

where ${T}_{{ss}}$ is the super-solidus, ${T}_{s}$ is the solidus temperature, and ${T}_{l}$ is the liquidus temperature. The solidus and liquidus for the crust and mantle are both temperature- and pressure-dependent and are parameterized by a polynomial relationship between temperature and pressure^88^ as

16 $${T}_{s}={a}_{s}+{b}_{s}P+{c}_{s}{P}^{2},$$

17 $${T}_{l}={a}_{l}+{b}_{l}P+{c}_{l}{P}^{2},$$

where *a*, *b*, and *c* are constants and are defined in Supplementary Table 4. The parameters mentioned in Supplementary Table 4 for calculating melt fraction were derived from Rey and Muller^89^ and Korchinski et al.^86^. As such, our final estimate of material density is dependent on melt fraction and temperature. Therefore, the density change caused by melt fraction expansion factor (*γ* = 0.13)^86^ and the fraction of melt (${M}_{f}$) affect the final density evolution of materials in our model, and Eq. (13) becomes

18 $$\rho (T,F)={\rho }_{0}\left[1-\left(\beta \delta T\right)-\left(\gamma F\right)\right].$$

In the case of Newtonian flow, the system usually requires a few iterations to converge and result in a truly incompressible flow based on the implemented iterative scheme and the chosen value for tolerance. In the case of non-Newtonian flow, where an effective viscosity depends on the velocity field through the strain rate, pressure, and temperature, the convergence of the computed fields in UWGeodynamics is achieved by nonlinear Picard-type iterations^90^ that result in a truly incompressible flow with the implemented iterative scheme. We define a maximum of 30 Picard-type nonlinear iterations, and the stopping criterion for the iteration process is determined based on a nonlinear tolerance of 0.02 for the solutions to the Stokes equations.

To ensure stable convergence, the time stepping (d*t*) used for time integration of the mechanical part in UWGeodynamics complies with Courant–Friedrichs–Lewy condition^91^ which is a first-order forward Euler algorithm as

19 $${{{{\rm{d}}}}t}={\min }\left(\frac{\Theta }{\left|u\right|},\frac{{\Theta }^{2}}{\alpha }\right)\times C,$$

where Θ is the smallest element/grid size, *α* is the maximum thermal diffusivity, |*u*| is the absolute maximum velocity, and *C* is the Courant number, greater than 0 and smaller than 1, to ensure an efficient and temporally stable model.

### Mantle convection simulation

We use the open-source finite-element code ASPECT^43–45^ (short for Advanced Solver for Problems in Earth ConvecTion) to solve the equations for conservation of momentum, energy, and mass, assuming incompressible Stokes flow and the extended Boussinesq approximation:

20 $$\nabla \cdot u=0,$$

21 $$\rho {C}_{p}\left(\frac{\partial T}{\partial t}+u\cdot \nabla T\right)=\nabla \cdot \left(k\nabla T\right),$$

22 $$\nabla \cdot (2\eta \dot{\epsilon })-\nabla p=-\rho g,$$

where *u* is the velocity, *T* is the temperature, *t* is time, *ρ* is the density, $\rho ={\rho }_{0}[1-\alpha \left(T-{T}_{0}\right)]$ with ${T}_{0}$ the reference temperature (1600 K), ${\rho }_{0}$ the reference density (3300 kg/m^3^), and *α* the coefficient of thermal expansion (3e^−5^ K^−1^), ${C}_{p}$ is specific heat capacity (1250 J/K kg), k is thermal conductivity (0 W m^−1 ^K^−1^), *η* is the viscosity, $\dot{\epsilon }$ is the deviator of the strain rate tensor $\dot{\epsilon }=\frac{1}{2}(\nabla u+{(\nabla u)}^{T})$, $\nabla \cdot (2\eta \dot{\epsilon })$ is the stress gradient, ∇*p* is the pressure gradient, and *ρg* is the driving force.

The domain of our global mantle convection simulation is a 3-D spherical shell. In our simulations, we use an ‘initial global refinement’ parameter of 4 so that the finite-element mesh contains 12 × (32)^3^= 393,216 cells. We apply free-slip boundary conditions at the surface and core–mantle boundary, and we remove the net rotation component of the flow solution. Using the global density distribution model TX2008^17,40^ we simulate the temporal evolution of global mantle flow by backward advecting density perturbations, assuming no diffusion. Present-day temperatures are obtained from the TX2008 density field through thermal expansion. Boundary temperatures are 1600 K and 3300 K at the surface and core–mantle boundary, respectively. Our convection simulation incorporates Newtonian rheology with a 1-D viscosity profile from Moucha et al.^17^. We determine estimates of spatial and temporal variations in deviatoric stress tensors and the associated traction field at different depths from the time-dependent mantle convection model.

#### Surface processes simulation

We use an open-source landscape evolution modeling code, Badlands^92^, to simulate the evolution of topography, sediment erosion, transport, and deposition, as well as the evolution of paleo-stream path over time. The governing equations in Badlands contain two main surface processes of fluvial incision and hillslope diffusion, which are described by geomorphic equations and explicitly solved with Badlands code^92^. The Badlands model is based on several simplifying assumptions associated with modeling surface processes such as the spatial and temporal uniformity of soil properties (e.g. particle size and bulk density) as well as non-differentiation between regolith and bedrock^92^. Therefore, the mass continuity in Badlands is determined based on the interactions among (1) tectonic forces, (2) long-term slope-driven diffusive processes determined by a diffusion law, and (3) fluvial processes defined as a stream-power law^92^. The capability of Badlands for simulating continental-scale landscape evolution over tens of millions of years makes it a suitable numerical tool to explore the landscape evolution of southwestern North America and quantify the interactions between surface evolution, tectonic forcing, and sediment distribution under different geodynamic histories of mantle and lithospheric processes.

In Badlands, the continuity of mass is defined by

23 $$\frac{{{{{\rm{d}}}}Z}}{{{{{\rm{d}}}}t}}=-\nabla \cdot {q}_{s}+u,$$

where *u* is the uplift rate (m yr^–1^), and ${q}_{s}$ is downhill sediment transport per unit width (m^2^ yr^–1^). The downhill sediment transport (${q}_{s}$) can be represented as a combination of incorporating sediment transport by (1) channel flow $({q}_{r})$, which is described by a stream power-law, and (2) long-term slope-driven diffusive processes $({q}_{d})$, described by simple creep^93^. The erosional behavior and long-term evolution of fluvial systems in our simulation are based on a detachment-limited erosion model for river incision, which is resistant to erosion^94,95^, and is based on a single-flow-direction algorithm where water is routed over the land surface following the steepest direction of descent direction^95,96^. Mathematical representation of erosion by fluvial processes for a detachment-limited model follows a stream power-law, which is a simplified form of the usual expression of sediment transport by water flow, in which the erosion rate is a function of drainage area (*A*), net precipitation (*P*), and land-surface slope (∇*Z*)^93^:

24 $$-\nabla \cdot {q}_{r}=-{k}_{d}{({PA})}^{m}{(\nabla Z)}^{n},$$

where ${k}_{d}$ is a dimensional coefficient of erodibility of the channel bed, *m* and *n* are dimensionless empirically derived constants of erosion exponents for the shear stress exerted on channel beds (which are generally positive with the $\frac{m}{n}$ ratio of ~0.5^94^), *PA* is a proxy for water discharge that numerically integrates the total area and precipitation from upstream connected nodes, and *∇Z* is land surface slope.

The surficial layer (regolith) long-term transport processes, or hillslope processes, are simulated based on a linear diffusion law known as soil creep^94,95,97^, which states that the flux of sediment is proportional to the gradient of topography:

25 $$-\nabla \cdot {q}_{d}=-{k}_{{hl}}{\nabla }^{2}Z,$$

where ${k}_{{hl}}$ is the diffusion coefficient with different values for terrestrial and marine environments, and *z* is elevation. Both ${k}_{d}$ and ${k}_{{hl}}$ depend on lithology, precipitation, and channel hydraulics^98^ and are scale dependent^99^. Values of these model parameters are listed in Supplementary Table 5.

These geomorphic equations for fluvial incision and hillslope diffusion are solved in Badlands using a triangular irregular network^100^ (TIN), and a finite-volume method is used for defining the continuity equation^101^.

We simulate a continental-scale source-to-sink system for southwestern North America since the late Eocene for each 0.1 Myr time increment. The source-to-sink process in Badlands follows the conservation of mass such that the amount of mass associated with erosion equals that of deposition plus outflow^102^. The sediment accumulation in Badlands is a function of three important conditions: (1) existence of a depression area based on Planchon and Darboux^103^ algorithms, (2) a land surface located below sea level, and (3) a local elevation that is less than the aggregational slope.

Our time-dependent modeling of sediment transport from source to sink in Badlands is constrained by external forcing mechanisms such as 3-D lithospheric tectonics (subsidence, uplift, and horizontal displacements), climate (rainfall regimes), and sea-level fluctuations. We simulate erosion, transport, and deposition through a forward-modeling approach starting at the late Eocene. The duration of each forward-model run is 36 Myr with variable tectonics, precipitation, and eustasy, and our starting paleo-elevation model has a resolution of 10 km × 10 km (approximately ${0.1}^{^\circ }$ × ${0.1}^{^\circ }$). To simulate the impact of sea-level fluctuations through geological time, we define the sea-level evolution within Badlands using a published eustatic curve by Haq et al.^104^. To account for temporal variations in precipitation, we use reconstructed paleo-precipitation models to quantify the coupled evolution of precipitation patterns based on topographic variation over time. As such, with these external forcings, we can quantify the relative importance of climate, erosion, and tectonics.

### Reconstructed paleo-climate (precipitation) simulation

We carried out two sets of simulations to sample climate responses to variations of CO_2_, ice sheet, and SWNA topography across several key intervals of the Miocene with the Community Earth System Model version 2 (CESM2)^105^. Two fully coupled atmosphere-ocean-land simulations were developed to sample two end members of Miocene climate states: a quasi-greenhouse state with limited and unstable Antarctic ice sheet of early to middle Miocene (18–14 Ma)^106,107^, and an icehouse state with more expansive and stable eastern Antarctic ice sheet (~14 Ma and onward)^107^. Due to the computational costs, these simulations are carried out at 1.9 × 2.5° horizontal resolution of the atmosphere and land, and 1° of the ocean and sea ice, featuring 400 ppm and 284.7 ppm CO_2_ separately. These two end members provide separate constraints of ocean heat convergence, a quantity featuring the net influence of ocean circulation on surface energy budget, for another set of 9 simulations at a higher atmosphere and land resolution of 0.9 × 1.25°. These 9 simulations (Supplementary Fig. 1) were carried out with the mixed layer ocean component of the CESM2, sampling combined changes of CO_2_ and SWNA topography from 28 to 5 Ma (Supplementary Table 6). CO_2_ levels are inferred from the published Miocene time series by Kurschner et al.^108^. Paleo-topography estimates of southwestern North America are derived from the model of Bahadori and Holt^10^. For simulations featuring CO_2_ and topography from 28 to 15.5 Ma, we use ocean heat convergence from the simulated mid-Miocene state. For simulations featuring CO_2_ and topography from 14 to 5 Ma, we use ocean heat convergence from the simulated icehouse state.

Both coupled simulations were run for 2000 model years. The top of the atmosphere energy imbalance reached a small value of ~0.2 W/m^2^, suggesting quasi-equilibrium. Both simulations show reasonable skill at capturing the general climate structure as characterized by the zonal sea surface and terrestrial temperature gradients of the mid-Miocene and late Miocene, estimated using Miocene proxy data compilations reported by Burls et al.^109^ (Supplementary Fig. 2). CESM2 simulations tend to overestimate tropical temperatures, and subsequently, the temperature gradient between subtropics and tropics. This mismatch, however, does not extend to the mid-latitudes, for which our surface processes simulations are conducted, and proxy and simulated temperatures show a good match there. The paleo-climate models produce estimates of precipitation in SWNA that are used as forcing conditions in surface processes model. This is how the coupling of lithosphere and climate is implemented.

### Macrostrat data reconstruction

We analyzed sedimentary packages from Macrostrat data^48^ for southwestern North America from the late Eocene to the present by performing a reconstruction for the present-day coordinates of recorded sedimentary packages based on the method and solution for southwestern North America found in Bahadori et al.^9^. While each stratigraphic column may include a combination of sedimentary, volcanic, and plutonic deposits, we considered maximum thicknesses as well as top and base ages associated with only sedimentary packages from the Macrostrat data. We then calculated sediment accumulation rates for each stratigraphic column (specific coordinates) for time bins of 0.1 Myr. The cumulative sediment accumulation through time is determined by adding together calculated sediment accumulation rates for each 0.1 Myr up to the present day.

## Supplementary information

Supplementary Information

Description of Additional Supplementary Files

Supplementary Movie 1

Supplementary Movie 2

Supplementary Movie 3

Supplementary Movie 4

Supplementary Movie 5

## Data Availability

The crustal structure, temperatures, volcanic, and metamorphic core complex data can be accessed at https://pubs.geoscienceworld.org/gsa/geosphere/article/14/3/1207/530582/ Reconstruction-modeling-of-crustal-thickness-and and https://www.nature.com/articles/s41467-019-12950-8#Sec14. The input files to reproduce the data that support the findings in this study have been deposited in the GitHub repository https://github.com/alireza-bahadori/paper-Coupled-influence-of-tectonics-climate-and-surface-processes.

## Code availability

The original version of the codes used to perform the simulations in this study can be accessed at https://github.com/badlands-model/badlands, https://github.com/underworldcode/UWGeodynamics, https://github.com/ESCOMP/CESM, and https://aspect.geodynamics.org. The codes used to make the figures can be accessed at www.soest.hawaii.edu/gmt/ and www.paraview.org/. The repository https://github.com/alireza-bahadori/paper-Coupled-influence-of-tectonics-climate-and-surface-processes contains the input files to reproduce the model results, as well as information on the UWGeodynamics installation and model setup performed in this study.
