# Supplementary material for: Coupled influence of tectonics, climate, and surface processes on landscape evolution in southwestern North America
Source: Nat Commun. 2022 Aug 1;13:4437. doi: 10.1038/s41467-022-31903-2 (PMC9343645; doi:10.1038/s41467-022-31903-2)
Supplement: Supplementary file 1 — Supplementary Information [file 41467_2022_31903_MOESM1_ESM.pdf]

Supplementary Information

for

Coupled influence of tectonics, climate, and surface processes on landscape evolution in southwestern North America

Alireza Bahadori[1,2*], William E. Holt[2], Ran Feng[3], Jacqueline Austermann[1], Katharine M. Loughney[4], Tristan Salles[5], Louis Moresi[6], Romain Beucher[6], Neng Lu[6], Lucy M. Flesch[7], Christopher M. Calvelage[7], E. Troy Rasbury[2], Daniel M. Davis[2], Andre R. Potochnik[8], W. Bruce Ward[9], Kevin Hatton[2], Saad S. B. Haq[7], Tara M. Smiley[10], Kathleen M. Wooton[2], Catherine Badgley[11]

[1]Lamont-Doherty Earth Observatory, Columbia University in the City of New York, Palisades, NY, USA. [2]Department of Geosciences, Stony Brook University, Stony Brook, NY, USA. [3]Department of Geosciences, University of Connecticut, Storrs, CT, USA. [4]Department of Geology, University of Georgia, Athens, GA, USA. [5]School of Geosciences, University of Sydney, Sydney, New South Wales, Australia. [6]Research School of Earth Sciences, The Australian National University, Canberra, Australia. [7]Department of Earth, Atmospheric, and Planetary Sciences, Purdue University, West Lafayette, IN, USA. [8]Grand Canyon Conservancy Field Institute, Flagstaff, AZ, USA. [9]e4sciences, Sandy Hook, CT, USA. [10]Department of Ecology and Evolution, Stony Brook University, Stony Brook, NY, USA. [11]Department of Ecology and Evolutionary Biology, University of Michigan, Ann Arbor, MI, USA. *Corresponding author: abahadori@ldeo.columbia.edu

Supplementary Tables

Supplementary Table 1 | Model parameters applied in thermo-mechanical model.

Material	Density (kg/m^3)	Heat capacity ($J/^{\circ}K.kg$)	Heat diffusivity (m^2/s)	Radiogenic heat production (MW/m^3)	Flow law (dislocation creep)
Topography	2500	1000	8.3×10^{-7}	0.5	Wet Quartz (Gleason and Tullis, 1995)
Upper crust	2700	1000	8.3×10^{-7}	0.9	Wet Quartz (Gleason and Tullis, 1995)
Middle crust	2900	1000	6.7×10^{-7}	0.9	Wet Diorite (Carter and Tsenn, 1987)
Lower crust	2900	1000	6.7×10^{-7}	0.9	Wet Diorite (Carter and Tsenn, 1987)
Mantle lithosphere	3330	1000	1.0×10^{-6}	0.022	Wet Olivine (Burov, 2011)
Mantle asthenosphere	3400	1000	1.0×10^{-6}	0.022	Dry Olivine (Karato and Wu, 1993)
Oceanic crust	2950	1000	1.0×10^{-6}	0.5	Dry Maryland Diabase (strong) (Burov, 2011)
Colorado Plateau lower crust	2900	1000	6.7×10^{-7}	0.9	Dry Maryland Diabase (strong) (Burov, 2011)
Colorado Plateau middle crust	2900	1000	6.7×10^{-7}	0.9	Wet Quartz (Gleason and Tullis, 1995)
Trench	2700	1000	1.0×10^{-6}	0.5	Wet Diorite (Carter and Tsenn, 1987)
Sediment	2300	1000	1.0×10^{-6}	0.6	Wet Quartz (Gleason and Tullis, 1995)
Sticky air	1000	100	1.0×10^{-6}	0	1.0×10^{19} Pa s

Supplementary Table 2 | Model parameters of plasticity applied in thermo-mechanical model.

Parameter	Value
Cohesion of pristine material (C_0)	10.0 MPa
Cohesion after softening (C_∞)	2.0 MPa
Friction coefficient of pristine material (μ_0)	0.1
Friction coefficient after softening (μ_∞)	0.01
Strain range of softening (ϵ)	0.0-0.5

Supplementary Table 3 | Model parameters of rheology applied in thermo-mechanical model.

Flow law (dislocation creep)	A_0 (Mpa^n/S)	n	E_0 (kJ/mole)	V_0 ($m^3/mole$)	R(J/mole·K)	f
Wet Diorite	3.2×10^{-2}	2.4	212	0	8.3144	*tr: $5e^{-4}$; lc: 25; mc: 2
Wet Olivine	275.6	4.45	498	0	8.3144	*ml: 0.4
Dry Olivine	4.8	3	502	0	8.3144	*ma: 1.75
Dry Maryland Diabase	8	4.7	485	0	8.3144	*oc: 1; lccp: 1
Wet Quartz	1.1×10^{-28}	4	223	3.1×10^{-6}	8.3144	*uc: $7.5e^{-3}$, mccp:1.5, se: 1

*uc: upper crust; mccp: middle crust Colorado Plateau; se: sediment; oc: oceanic crust; lccp: lower crust Colorado Plateau; tr: trench; lc: lower crust; mc: middle crust; ml: mantle lithosphere; ma: mantle asthenosphere.

Supplementary Table 4 | Model parameters of partial melting processes applied in thermo-mechanical model.

**Parameter**	**Value**
Latent heat of fusion (kJ/kg)	250
Melt fraction density change (k)	0.13
Solidus coefficient ‘a_s’ (k)	993
Solidus coefficient ‘b_s’ (k/Pa)	$-1.2e^{-7}$
Solidus coefficient ‘c_s’ (k/Pa)	$1.2e^{-16}$
Liquidus coefficient ‘a_l’ (k)	1493
Liquidus coefficient ‘b_l’ (k/Pa)	$-1.2e^{-7}$
Liquidus coefficient ‘c_l’ (k/Pa^2)	$1.6e^{-16}$
Melt viscous softening factor	$1.0e^{-2}$
Viscous softening melt fraction	0.15-0.3

Supplementary Table 5 | Model parameters applied in surface processes simulation.

**definition**	**Value**	**Symbol (unit)**
Exponent in stream-power law	0.5	m
Exponent in stream-power law	1.0	n
Erosion coefficient of fluvial process	4×10^{-7}	K_d (yr^{-1})
Terrestrial diffusion coefficient for hillslope processes	1	K_{hl}(m^2 yr^{-1})
Marine diffusion coefficient for hillslope processes	1	K_{hl} (m^2 yr^{-1})

Supplementary Table 6 | Time slices, CO_2 levels, and southwestern North America paleo-topography featured by the experiments.

**Time**	**CO_2 level**	**The key Miocene topographic evolution of southwestern North America**
28 Ma	341 ppm	Collapse of the northern Nevadaplano and southern Mogollon Highlands
21 Ma	341 ppm	Collapse of the northern Nevadaplano and northern Mogollon Highlands
19 Ma	410 ppm	Collapse of the northern Nevadaplano and northern Mogollon Highlands
17 Ma	374 ppm	Collapse of the southern Nevadaplano
15.5 Ma	440 ppm	Collapse of the southern Nevadaplano
14 Ma	284.7 ppm	Renewed topographic collapse of the entire Nevadaplano
10.5 Ma	374 ppm	Renewed topographic collapse of the entire Nevadaplano
8 Ma	284.7 ppm	Continued extension
7 Ma	341 ppm	Continued extension
6.5 Ma	284.7 ppm	Continued extension

Supplementary Figures

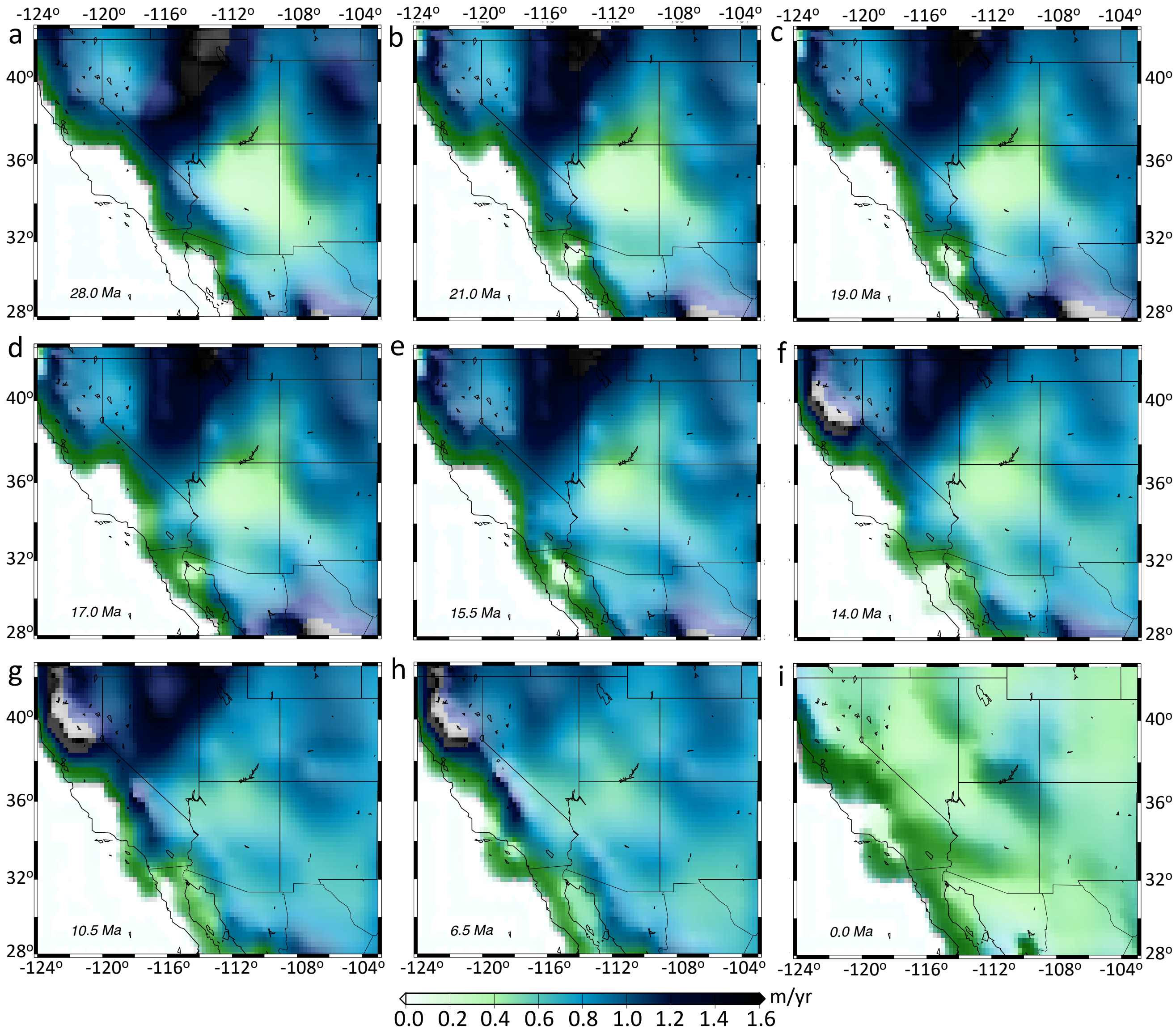

Supplementary Figure 1 | Reconstructed paleo-climate (precipitation) models for southwestern North America since the late Eocene. a-i, Variations in precipitation rate in southwestern North America at 28, 21, 19, 17, 15.5, 14, 10.5, 6.5, and 0 Ma, respectively. The map images were created by authors using: www.soest.hawaii.edu/gmt/ and www.paraview.org/.

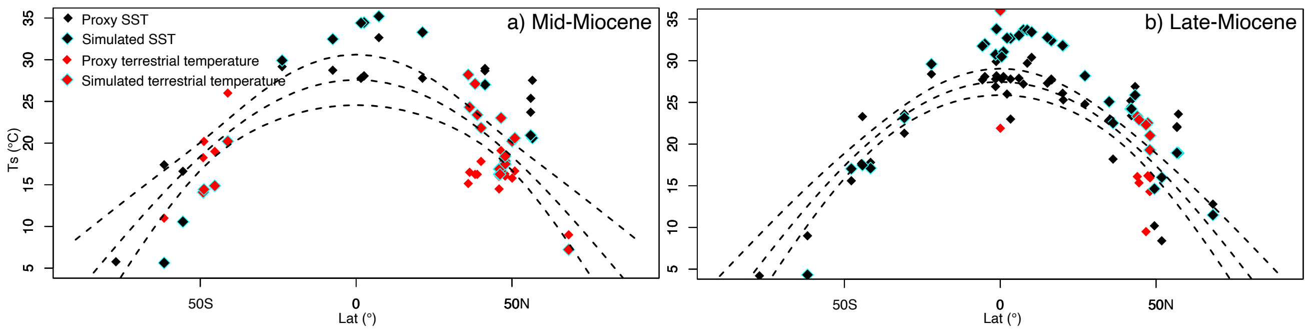

Supplementary Figure 2 | Proxy-model comparison of meridional distributions of sea surface and terrestrial surface temperatures. a-b, The dashed lines are least square fit and 95% confidence interval of proxy meridional surface temperature as a function of cosine latitude. Marks are proxy temperatures and model temperatures at the proxy sites. SST: sea surface temperature.

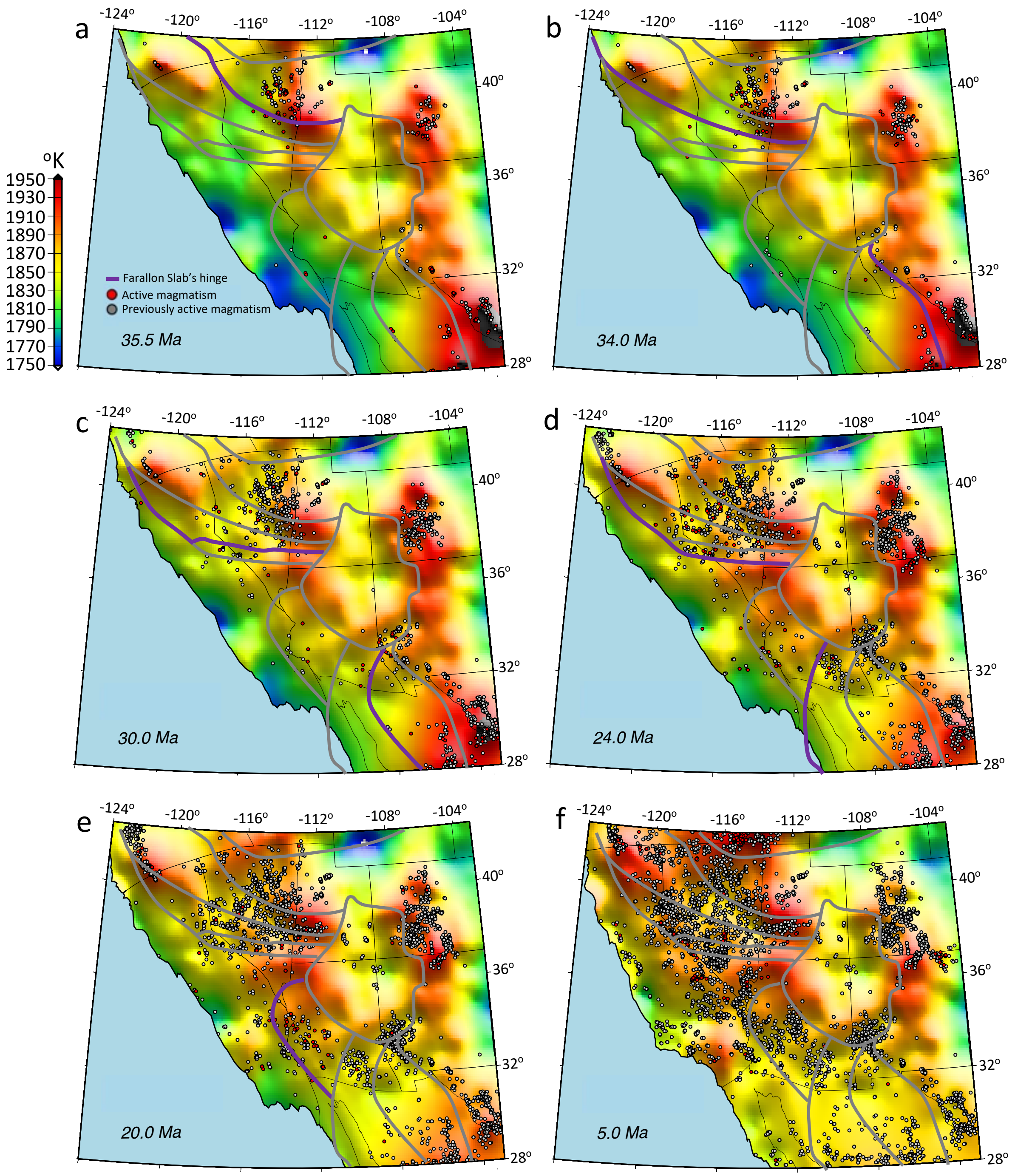

Supplementary Figure 3 | Paleo-magmatism evolution associated with the Farallon Slab rollback in southwestern North America since the late Eocene. a, Timing and placement of Cenozoic magmatic patterns from Bahadori and Holt (2019) and the inferred temperature changes at 200 km depth in southwestern North America at 36 Ma. The closed gray line represents the present-day boundary of the Colorado Plateau. Red dots represent active paleo-magmatism for the time shown in each panel, gray dots represent previously active paleo-magmatism, gray lines represent the location of the Farallon Slab's hinge during slab rollback from Dickinson (2002), and purple lines represent the location of the Farallon Slab's hinge for the time shown in each panel; **b-f,** Similar to 'a' but at 34, 30, 24, 20, and 5 Ma. Palinspastic reconstruction of state boundaries is from Bahadori et al. (2018). The map images were created by authors using: www.soest.hawaii.edu/gmt/ and www.paraview.org/.

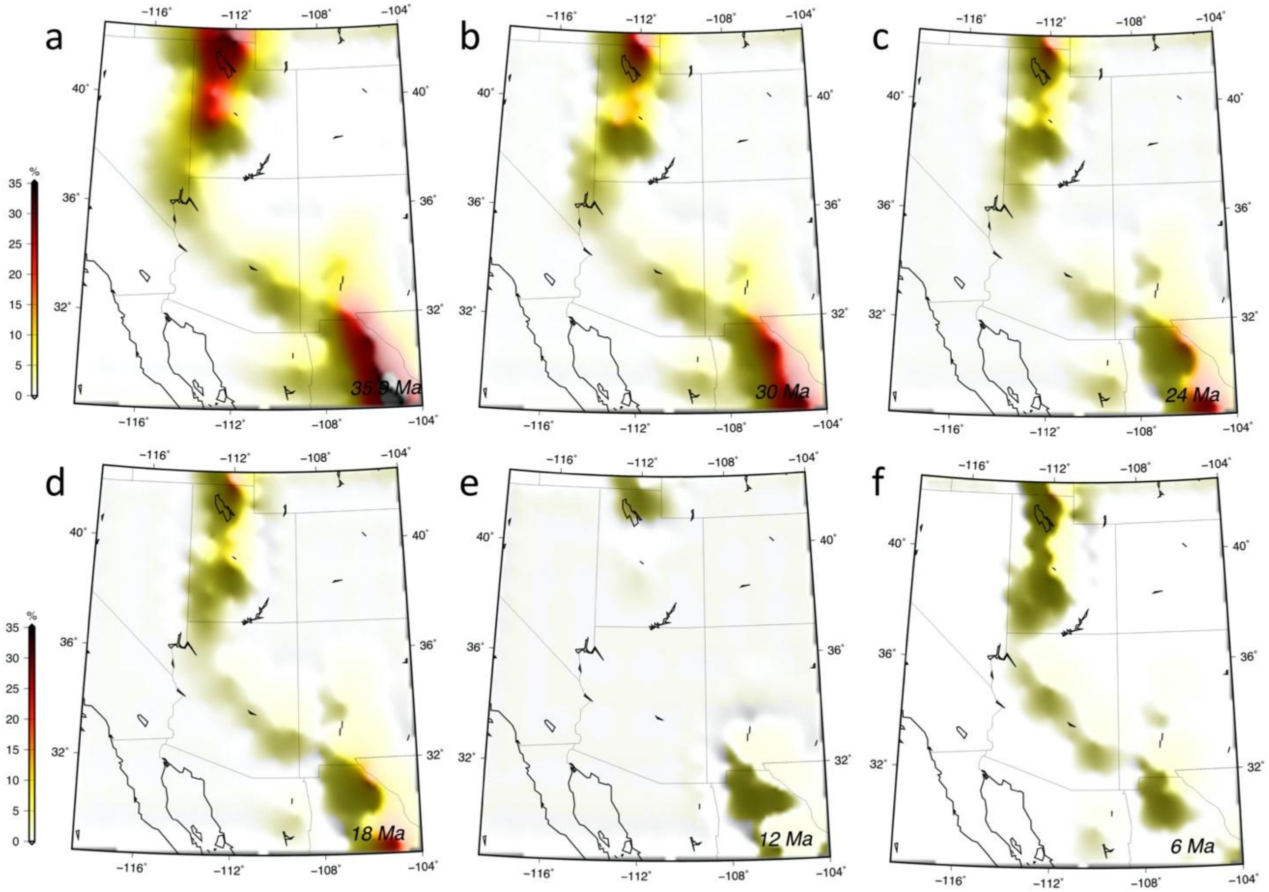

Supplementary Figure 4 | The partial melting of the lower crust. a-f, Variation of partial melt magnitude within lower crust of the Basin and Range Province at 35.8 Ma, 30.0 Ma, 24 Ma, 18 Ma, 12 Ma, and 6 Ma, respectively. The map images were created by authors using: www.soest.hawaii.edu/gmt/ and www.paraview.org/.

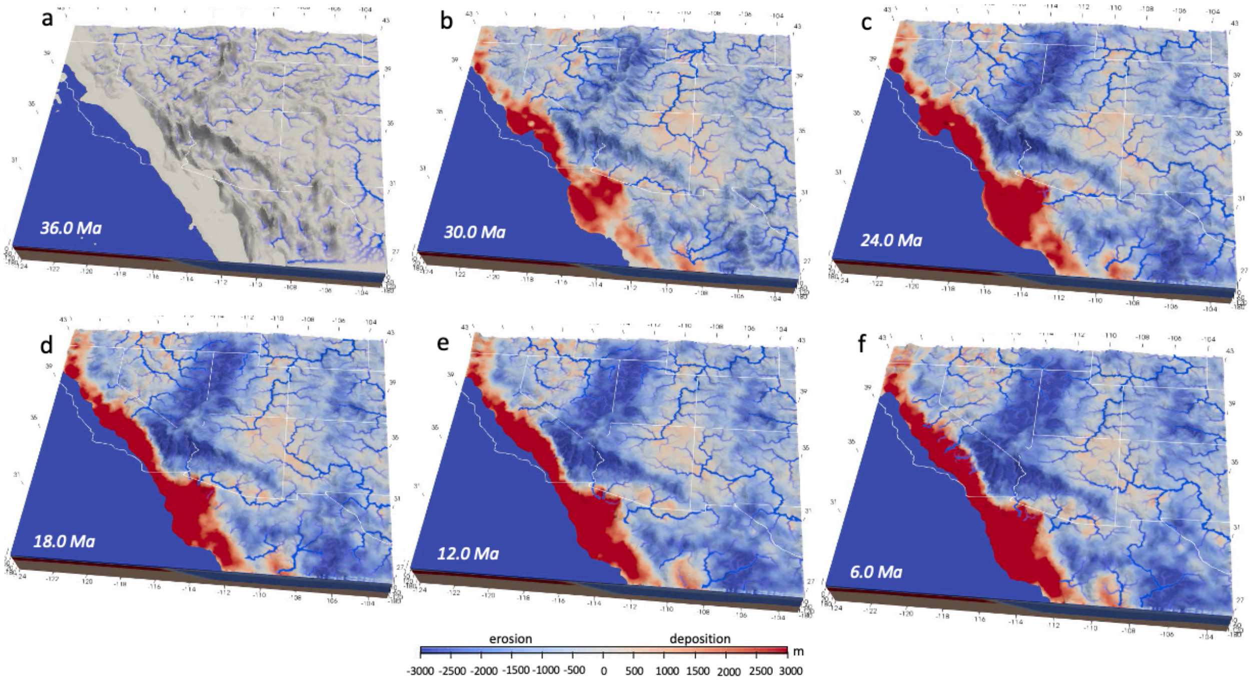

Supplementary Figure 5 | Interaction and interconnection between tectonics, climate, and surface processes. a-f, Evolution of drainage, erosion, and sediment accumulation in southwestern North America from the surface processes model. The map images were created by authors using: www.soest.hawaii.edu/gmt/ and www.paraview.org/.

Supplementary References:

Bahadori, A., Holt, W. E. & Rasbury, E. T. Reconstruction modeling of crustal thickness and paleotopography of western North America since 36 Ma. *Geosphere* **14**, 1207–1231 (2018).

Bahadori, A. & Holt, W. E. Geodynamic evolution of southwestern North America since the Late Eocene. *Nat. Commun.* **10**, 1–18 (2019).

Dickinson, W. R. The Basin and Range Province as a composite extensional domain. *Int. Geol. Rev.* **44**, 1–38 (2002).
