# Supplementary material for: Coupled influence of tectonics, climate, and surface processes on landscape evolution in southwestern North America
Source: Nat Commun. 2022 Aug 1;13:4437. doi: 10.1038/s41467-022-31903-2 (PMC9343645; doi:10.1038/s41467-022-31903-2)
Supplement: Supplementary file 2 — Description of Additional Supplementary Files [file 41467_2022_31903_MOESM2_ESM.pdf]

## Description of Additional Supplementary Files

File name: Supplementary Movie 1

Description: Radial cross-section of the reconstructed mantle temperature variations from surface to core-mantle boundary (CMB) at latitude 37$^{o}$N. Superimposed on the cross-section are the corresponding mantle flow velocity vectors. The vectors are extracted from global flow field in the mantle's no-net rotation frame of reference along a cross section fixed relative to North America.

File name: Supplementary Movie 2

Description: Evolution of the traction field associated with mantle convection at 200 km depth.Vectors represent horizontal components of the traction vector field at 200 km depth, and contours represent the radial component of the traction vector field.

File name: Supplementary Movie 3

Description: Temperature variations at 200 km depth used in thermo-mechanical modeling of the lithosphere. Red arrows represent kinematic field associated with sub-lithosphere tractions from mantle convection at 200 km depth.

File name: Supplementary Movie 4

Description: Variation of the principal axes of horizontal deviatoric stresses for the upper crust of the North American lithosphere. Black arrows represent compressional and red arrows represent tensional stresses. The color-coded circles and vectors represent the stretching direction of metamorphic core complexes shown in yellow, and Miocene faults and dykes trend shown in green and blue, respectively, from Bahadori and Holt (2019).

File name: Supplementary Movie 5

Description: Simulated paleo-elevation and drainage path in southwestern North America since thelate Eocene.
